# Decoupling body shape and mass distribution in birds and their dinosaurian ancestors

**DOI:** 10.1038/s41467-023-37317-y

**Published:** 2023-03-22

**Authors:** Sophie Macaulay, Tatjana Hoehfurtner, Samuel R. R. Cross, Ryan D. Marek, John R. Hutchinson, Emma R. Schachner, Alice E. Maher, Karl T. Bates

**Affiliations:** 1 Department of Musculoskeletal & Ageing Science, Institute of Life Course & Medical Sciences, University of Liverpool, William Henry Duncan Building, 6 West Derby Street, Liverpool, L7 8TX UK; 2 Department of Life Sciences, School of Life Sciences, University of Lincoln, Joseph Banks Laboratories, Green Lane, Lincoln, LN6 7DL UK; 3 Department of Cell & Development Biology, Division of Biosciences, University College London, Anatomy Building, Gower Street, London, WC1E 6BT UK; 4 Structure and Motion Laboratory, Department of Comparative Biomedical Sciences, Royal Veterinary College, Hatfield, AL9 7TA UK; 5 Department of Cell Biology and Anatomy, School of Medicine, Louisiana State University Health Sciences Center, New Orleans, LA 70112 USA

**Keywords:** Palaeontology, Biomechanics, Phylogeny

## Abstract

It is accepted that non-avian theropod dinosaurs, with their long muscular tails and small forelimbs, had a centre-of-mass close to the hip, while extant birds, with their reduced tails and enlarged wings have their mass centred more cranially. Transition between these states is considered crucial to two key innovations in the avian locomotor system: crouched bipedalism and powered flight. Here we use image-based models to challenge this dichotomy. Rather than a phylogenetic distinction between ‘dinosaurian’ and ‘avian’ conditions, we find terrestrial versus volant taxa occupy distinct regions of centre-of-mass morphospace consistent with the disparate demands of terrestrial bipedalism and flight. We track this decoupled evolution of body shape and mass distribution through bird evolution, including the origin of centre-of-mass positions more advantageous for flight and major reversions coincident with terrestriality. We recover modularity in the evolution of limb proportions and centre-of-mass that suggests fully crouched bipedalism evolved after powered flight.

## Abstract (web summary)

Here, the authors track the evolution of mass distribution through bird evolution challenging suggested coupling between body shape and centre-of-mass position, and instead showing that crouched bipedalism evolved after powered flight.

## Introduction

**Fig. 1:**
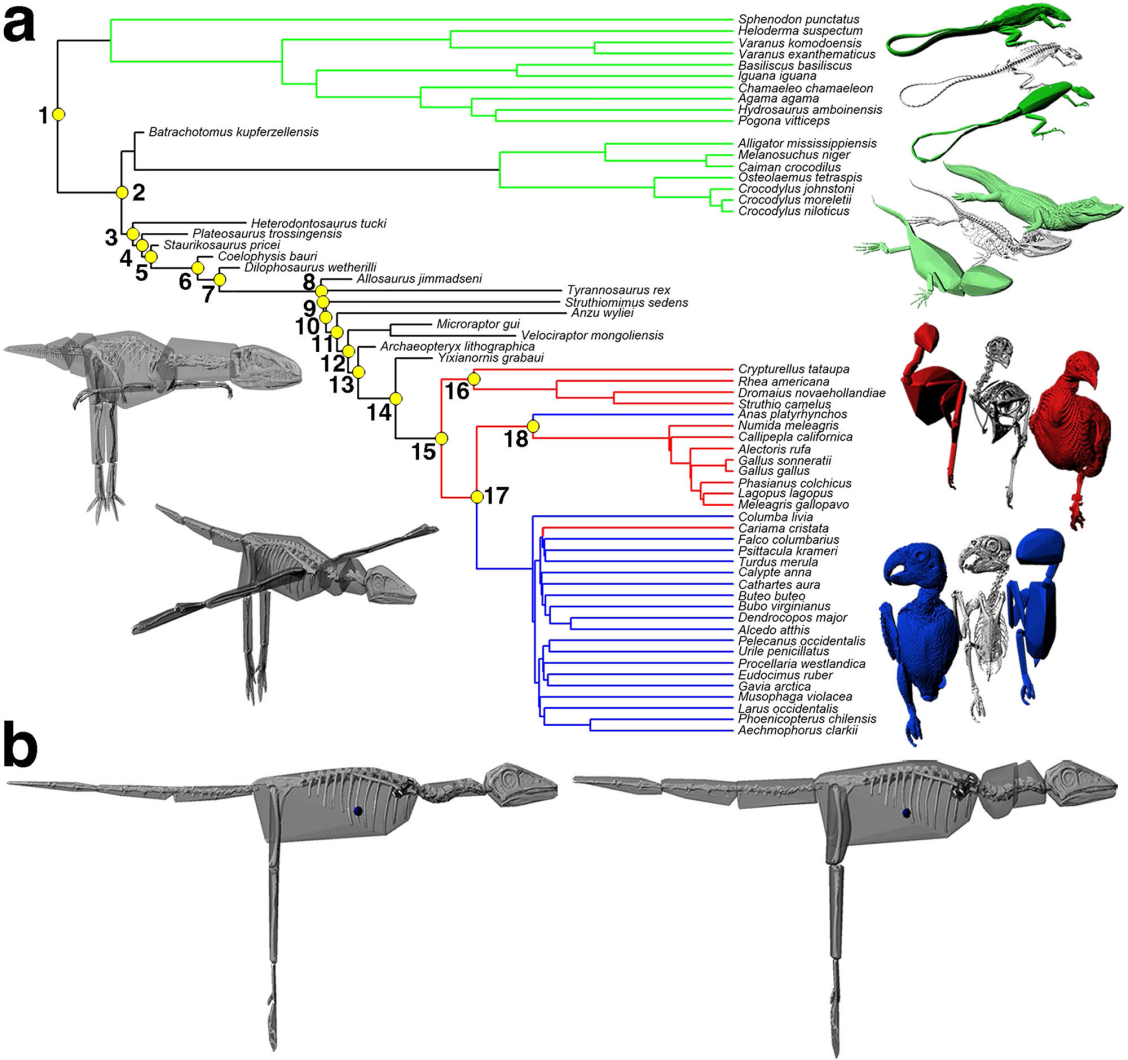
Reconstructing body proportions and centre-of-mass in bird-line archosaurs. **a** Supertree of all taxa in the study, with branch lengths scaled to unit time. The larger yellow circles represent the major reconstructed nodes through avian evolution, and are numbered as followed, 1. Sauropsida, 2. Archosauria, 3. Dinosauria, 4. Saurischia, 5. Theropoda, 6. Neotheropoda, 7. *Dilophosaurus* + Neotetanurae, 8. Neotetanurae, 9. Coelurosauria, 10. Maniraptoriformes, 11. Pennaraptora, 12. Eumaniraptora, 13. Avialae, 14. Ornithuromorpha, 15. Neornithes, 16. Palaeognathae, 17. Neognathae, 18. Galloanserae. Three dimensional skeletal, minimum skeletal convex hull and skin volume models were generated from CT scans of 17 extant non-avian sauropsids (green branches), 13 hindlimb-dominated (HLD; red branches) and 20 forelimb-dominated (FLD; blue branches) extant birds. These data were used to statistically assess associations between body proportions and locomotion in extant birds, and (**b**) to develop predictive relationships between minimum skeletal convex hulls and skin volume that could be applied to estimate segment and whole-body mass properties in archosaurian fossils, including those along the dinosaurian lineage leading to extant birds (black branches). In (**b**) the minimum skeletal convex hulls of *Archaeopteryx* (left image) have been expanded by the average expansion factors measured for individual body segments (right image) in the two extant phylogenetic bracket groups (non-avian sauropsids and birds), allowing calculation of the whole-body centre-of-mass position (blue spheres).

Newtonian mechanics dictates that body shape and mass distribution play fundamental roles in the physics and physiology of animal movement^1^. The lengths and masses of body segments influence the forces and energetics required to enact motion, and therefore it is expected that major transitions in locomotor mode should be coupled with adaptive modifications to body shape^2–9^. Recognition of theropod dinosaurs as the direct ancestors of birds^10^ revealed that the avian lineage underwent dramatic changes in body shape during its evolutionary history (Fig. 1), epitomised in the contrast between the long muscular tails and small forelimbs of Mesozoic theropods like *Compsognathus* and the highly reduced tails and large wings of extant flying birds. This change in body shape, tracked by skeletal fossils^6,7,11^, has led to various hypotheses about how mass distribution, or whole-body centre-of-mass (CoM), was adaptively modified in concert with body proportions during the evolution of birds^6–9^. These competing hypotheses vary in the specific predictions made about the timing of evolutionary changes, but fundamentally they share the same overarching paradigm: that the dinosaurian ancestors of birds had a CoM close to the hips, while modern birds have their mass centred more cranially.

The shift between these dichotomous body shapes and inferred mass distributions is considered central to the evolution of two key innovations in the avian locomotor system: crouched bipedalism and powered flight^6–9,11,12^. The location of the CoM is a major determinant of the limb posture in bipedal animals^13–16^. Extant Neornithes stand and move with an unusually flexed hip, placing the feet cranial to the hip and the knee tending to be cranial to the ground reaction force around midstance^16,17^. This mechanically challenging posture has been mechanistically linked to a more cranial CoM position in birds^6–9,12^ and is further facilitated by a series of osteological and muscular specialisations within the hindlimb^11,17–21^. Transition towards the more cranial ‘avian’ CoM position and crouched bipedalism has been inferred to have begun in early maniraptoran theropods^6,7,9^, with the close phylogenetic proximity to the evolution of powered flight suggesting that whole-body shape and mass distribution represents a link between the emergence of these two key innovations in the avian locomotor system^6–9,12^. However, while studies of mass distribution in extinct dinosaurs are commonplace^9,22–25^, relatively few studies have quantified CoM position in living birds. Skeletal proportions in modern birds vary enormously^5,11,26,27^ and this lack of comparative data on mass distribution substantially limits our understanding of how a major component of their morphological and phenotypic diversity relates to ecological variation, both across extant groups and relative to their dinosaurian ancestors.

In this study, we use new image-based volumetric models (Fig. 1) to challenge the current paradigm used to interpret the evolution of avian locomotion. We demonstrate that qualitative differences in mass distribution between theropod dinosaurs and modern birds do not exist, despite their obvious difference in overall body shape. This decoupling of body shape and mass distribution has important implications for interpretations of locomotor evolution in theropod dinosaurs and birds.

## Results

### CoM position, body segment proportions and locomotion in extant birds

**Fig. 2:**
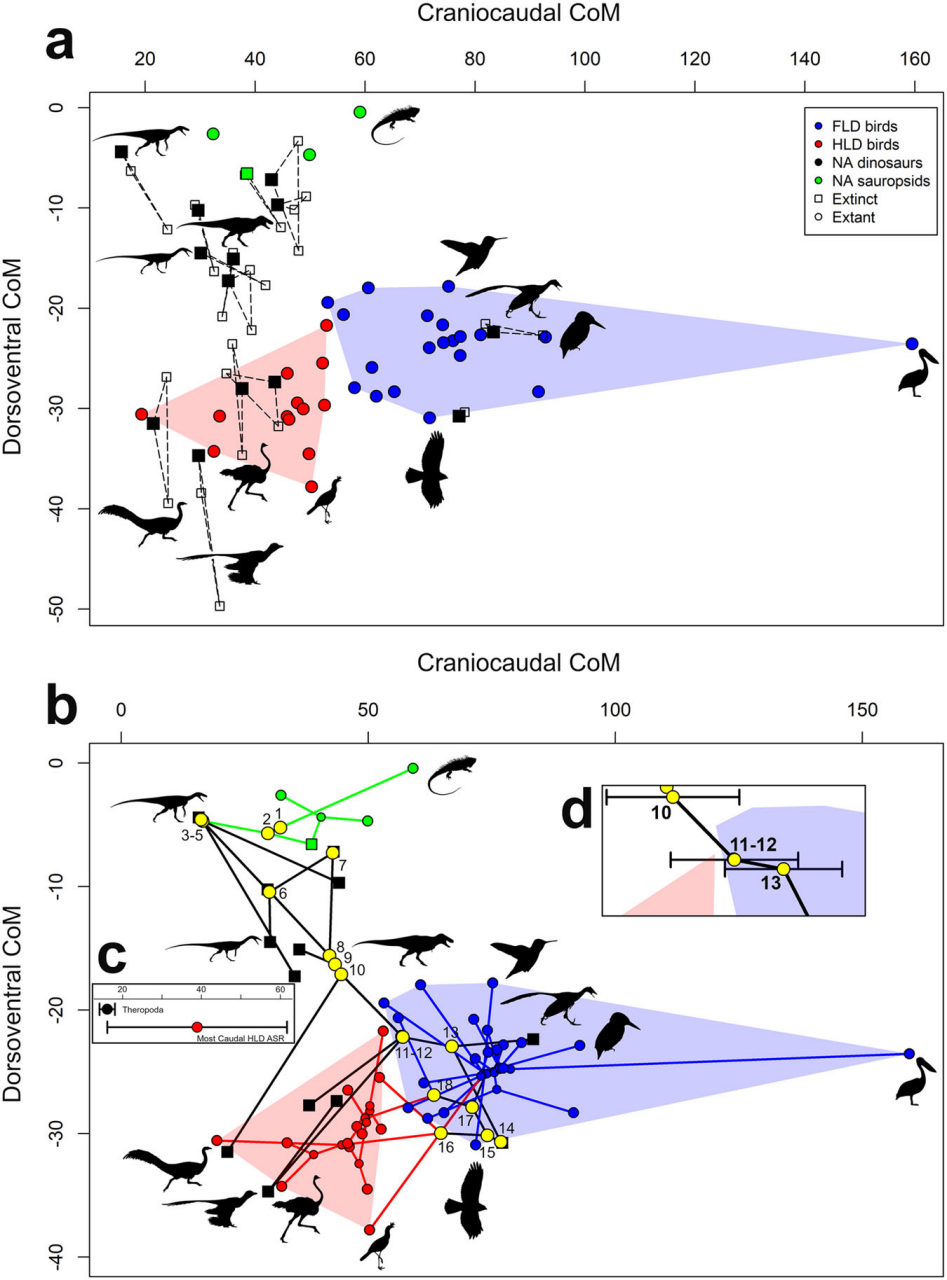
Centre-of-mass evolution in bird-line archosaurs. **a** Individual taxon normalised CoM positions (distance cranial and ventral to hip/body mass^0.33^) measured in extant birds and non-avian sauropsids, and predicted positions in extinct archosaurs based on skeleton:skin volume ratios and allometric equations from extant taxa. **b** CoM phylomorphospace plot of the 50 studied taxa, with extinct taxa represented as squares, and extant taxa (and surviving nodes) as circles. The larger yellow circles represent the major reconstructed nodes through avian evolution, and are numbered as followed, 1. Sauropsida, 2. Archosauria, 3. Dinosauria, 4. Saurischia, 5. Theropoda, 6. Neotheropoda, 7. *Dilophosaurus* + Neotetanurae, 8. Neotetanurae, 9. Coelurosauria, 10. Maniraptoriformes, 11. Pennaraptora, 12. Eumaniraptora, 13. Avialae, 14. Ornithuromorpha, 15. Neornithes, 16. Palaeognathae, 17. Neognathae, 18. Galloanserae. **c** Inset of the main plot (**b**) showing the overlapping CC_CoM confident intervals of the Theropoda node and those of the extant HLD bird node with the most caudal CoM position. **d** Inset of the main plot (**b**) showing the overlapping confidence intervals demonstrating that Avialae is first bird-line node to lie exclusively with extant FLD CoM morphospace. Green data points and lines represent extant non-avian sauropsids, black data points and lines are extinct non-avian sauropsids, red data points and lines represent hindlimb dominated birds, and blue data points and lines are forelimb dominated birds. Silhouettes of *Microraptor*, *Tyrannosaurus* and the ornithomimid by Matthew Dempsey, used with permission and without modification. Silhouettes of *Coelophysis* (CC BY 3.0; https://creativecommons.org/licenses/by/3.0/) and *Herrerasaurus* (CC BY 3.0; https://creativecommons.org/licenses/by/3.0/) by Scott Hartman sourced without modification from www.phylopic.org. Source data are provided as a Source Data file.

Hindlimb-dominated (HLD; predominantly terrestrial) birds are statistically different from forelimb-dominated (FLD; predominantly volant) birds in both their cranio-caudal CoM (CC_CoM) (*P* = 0.039; Supplementary Data 7) and dorso-ventral CoM (DV_CoM) positions (*P* = 0.012, Supplementary Data 7), with HLD birds having a more caudal and ventral CoM position (Fig. 2a. Supplementary Fig. 2). Removal of the pelican (which has the most extreme cranial CoM position in the data set; Fig. 2) had little effect on these relationships (Supplementary Data 7). HLD birds have greater body masses than FLD birds even when ratites and the pelican are removed, but in all three cases these differences are not statistically significant (Supplementary Data 8). Linear relationships between body mass and CoM positions are statistically significant across all birds, and within HLD and FLD groups (Supplementary Data 9–11). Across all birds and HLD birds, CC_CoM scales with negative allometry (Supplementary Data 9–10) indicating a relative caudal shift in CoM as body size increases. However, the upper 95% confidence intervals for the ‘all bird’ relationship does narrowly include isometry (Supplementary Data 9). In FLD birds this relationship is isometric, indicating no size-related change in CC_CoM position (Supplementary Data 11). Removal of ratites (the four largest taxa) from HLD birds results in an increase in group’s slope, but it remains negatively allometric (Supplementary Data 12), while removal of the pelican from the FLD group reduces the slope but 95% confidence intervals still include isometry (Supplementary Data 11). All categories exhibit slight positive allometry in their DV_CoM position, which indicates a small ventral shift in CoM as body size increases (Supplementary Data 9–12), with phylANCOVAs indicating there are no significant differences in slopes between locomotor groups, including when ratites and the pelican are removed (Supplementary Data 13). Correlations between raw taxon CoM positions and body segment proportions are provided in the Supporting Information and Supplementary Data 14–17.

pANOVAs indicate that FLD birds have significantly (*P* = < 0.05) greater skull lengths, shoulder widths, sternum depths, humeral lengths, forearm lengths, manus lengths, forelimb lengths and pes lengths, and significantly lower thigh lengths for their size than HLD birds (Supplementary Fig. 10, Supplementary Data 18). Differences between other parameters are not statistically significant (*P* = > 0.05). FLD birds also have significantly (*P* = < 0.05) greater head, humeral, hand and forelimb segment masses, and significantly lower shank and hindlimb masses for their size than HLD birds (Supplementary Fig. 11, Supplementary Data 19). Only humeral segment mass is impacted by removal of the pelican from the FLD data, with the difference becoming narrowly insignificant (*P* = 0.062).

### CoM and body segment evolution in bird-line archosaurs

Ancestral state reconstruction of CoM positions (Supplementary Data 22) recovers a caudal shift in CC_CoM position at Dinosauria (Fig. 2b), with a predicted CoM for this node and that of Theropoda marginally caudal to the range seen in extant HLD, but with 95% confidence intervals extending into that range (Fig. 2c). *Staurikosaurus* has the most caudal CoM position of the non-avian dinosaur taxa reconstructed here, and has two model iterations with a more caudal CC_CoM position, six model iterations within the range seen in HLD birds, and four that fall almost exactly on the caudal extreme of the HLD range (Fig. 2a, Supplementary Fig. 3a–c). The DV_CoM position of Dinosauria, Saurischia and Theropoda remain within the range seen in extant non-avian sauropsids (Fig. 2b). From Neotheropoda to Maniraptoriformes we recover a gradual cranial and ventral trend in CoM migration, with a minor dorsal retroversion at the *Dilophosaurus* + Neotetanurae node (Fig. 2b). Reconstructed CoM positions for the nodes Maniraptoriformes, Pennaraptora, and Eumaniraptora imply caudal and ventral shifts in CoM position within these lineages such that uncontroversially terrestrial taxa (*Struthiomimus*, *Anzu*, *Velociraptor*) plot within HLD bird CoM morphospace (Fig. 2b). The CC_CoM position of the Maniraptoriformes node plots within the range of HLD birds, while Pennaraptora is recovered at the caudal extreme of FLD bird CoM morphospace (Fig. 2b). However, the CC_CoM confidence intervals of these nodes bridge HLD and FLD bird CoM morphospace (Fig. 2d). Avialae is first bird-line node to lie exclusively within extant FLD CoM morphospace (Fig. 2b, d), with *Archaeopteryx* and *Yixianornis* plotting firmly within FLD CoM morphospace (Fig. 2a). Reconstructed ancestral states for Neornithes, Neognathae, and Galloanserae are located firmly within FLD CoM morphospace. Removal of the pelican from the data set had an extremely small quantitative effect on reconstructed ancestral states, and thus no qualitative effect on any of the aforementioned trends (Supplementary Figs. 5–6, Supplementary Data 22).

**Fig. 3:**
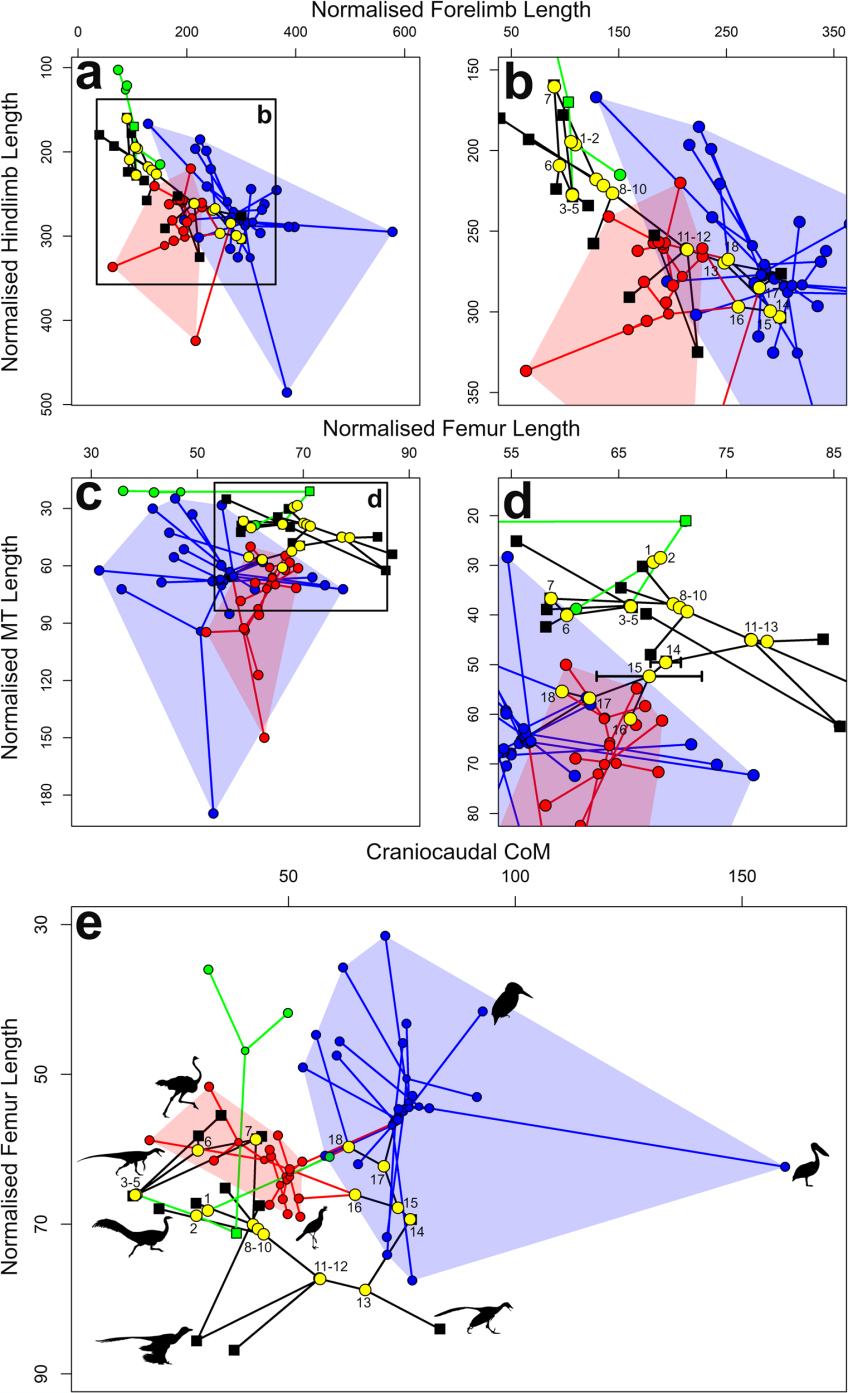
The evolution of relative limb proportions in bird-line archosaurs. Phylomorphospace plots of (**a**, **b**) normalised forelimb length and hindlimb length, (**c**, **d**) normalised femur length and metatarsal length, (**e**) normalised cranio-caudal CoM and normalised femur length in the 50 studied taxa, with extinct taxa represented as squares, and extant taxa (and surviving nodes) as circles. The larger yellow circles represent the major reconstructed nodes through avian evolution, and are numbered as followed, 1. Sauropsida, 2. Archosauria, 3. Dinosauria, 4. Saurischia, 5. Theropoda, 6. Neotheropoda, 7. *Dilophosaurus* + Neotetanurae, 8. Neotetanurae, 9. Coelurosauria, 10. Maniraptoriformes, 11. Pennaraptora, 12. Eumaniraptora, 13. Avialae, 14. Ornithuromorpha, 15. Neornithes, 16. Palaeognathae, 17. Neognathae, 18. Galloanserae. Green data points and lines represent extant non-avian sauropsids, black data points and lines are extinct non-avian sauropsids, red data points and lines represent hindlimb dominated birds, and blue data points and lines are forelimb dominated birds. Silhouettes of *Microraptor*, Silhouettes of *Microraptor*, *Tyrannosaurus* and the ornithomimid by Matthew Dempsey, used with permission and without modification. Silhouettes of *Coelophysis* (CC BY 3.0; https://creativecommons.org/licenses/by/3.0/) and *Herrerasaurus* (CC BY 3.0; https://creativecommons.org/licenses/by/3.0/) by Scott Hartman sourced without modification from www.phylopic.org. Source data are provided as a Source Data file.

Spearmans rank correlations suggest that the same body segments mostly exert qualitatively similar influences on CC_CoM trends across the whole data set (all nodes) and through the avian stem lineage (nodes 1–15 in Fig. 2b. See also Fig. 1, Supplementary Data 23–24): more cranial CC_CoM positions show strong statistically significant correlations with increases in forelimb segment lengths and masses, increasing shoulder width, skull and neck length, and reductions in tail length and mass (Supplementary Data 25, 27). Across the whole data set, the strongest correlations recovered are in the forelimb (e.g., forelimb length *Rho* = 0.937; forelimb segment masses *Rho* = 0.554–0.807, Supplementary Data 25), while through the avian stem lineage the tail is recovered with the strongest correlations (tail mass *Rho* = −0.989, tail length *Rho* = −0.950). Shank and metatarsal segment lengths show significant positive correlations through the avian stem lineage and all nodes. However, femur length shows a significant positive correlation through the avian stem nodes (i.e. more cranial CC_CoM correlated with longer femora), but a significant negative correlation across all nodes (i.e., more cranial CC_CoM correlated with shorter femora, Supplementary Data 25–27). This positive correlation is particularly strong between Neotetanurae and Avialae, with a noticeable reduction in relative femoral length occurring without any change in CC_CoM position occurring at Ornithuromorpha that realises a shift into FLD morphospace (Fig. 3e).

Statistically significant correlations are recovered between all segment masses (except the metatarsals segment) and DV_CoM across the whole data set (Supplementary Data 26), with torso mass (*Rho* = −0.736), neck mass (*Rho* = 0.748), tail mass (0.6016) and hindlimb mass (−0.590) yielding the strongest associations. The hindlimb (*Rho* = −0.623) and its more distal segments (shank length Rho = −0.512; metatarsal length Rho = −0.610) and the tail (*Rho* = 0.559) produce the strongest statistically significant correlations with DV_CoM among segment linear dimensions (Supplementary Data 26). Through the avian stem lineage, all body segment linear dimensions except shoulder width and pelvic and neck length show significant correlations with DV_CoM, with tail length (*Rho* = 0.921), forelimb length (*Rho* = −0.829) and pelvic width (*Rho* = 0.800) recovered with the strongest associations (Supplementary Data 28).

We also recover strong statistically significant positive correlations between hindlimb and forelimb lengths when all nodes are analysed (*rho* = 0.780, Supplementary Data 29), and particularly when only avian stem nodes are analysed (*rho* = 0.882, Supplementary Data 29, Fig. 3a, b). On a phylomorphospace plot of hindlimb and forelimb lengths, all non-avian nodes rootward to Pennaraptora plot outside extant bird morphospace owing to the combined effect of shorter hindlimbs and forelimbs (Fig. 3a, b). A shift into FLD phylomorphospace occurs at Avialae, primarily through elongation of the forelimb (Fig. 3a, b). In contrast, the shift into extant bird morphospace occurs at later-diverging nodes in femur-metatarsal length phylomorphospace (Fig. 3c, d). Palaeognathae is first node to lie exclusively within the shorter femora-longer metatarsal areas of morphospace occupied by modern birds, though the 95% confidence intervals of the Neornithes node overlaps with both HLD and FLD morphospace (Fig. 3c, d). Spearmans rank correlations indicate statistically significant associations between femur, shank and metatarsal segment lengths across all nodes in the analysis, with femur length negatively correlated with both shank and metatarsal length, and the latter two positively correlated with each other (Supplementary Data 29). The same qualitative switch in correlation that occurs in the relationship between femur length and CC_CoM (Fig. 3e) through the avian stem nodes versus all nodes (Supplementary Data 25, 27) also occurs in femur length versus shank length and metatarsal length (Supplementary Data 29), though these correlations do not reach statistical significance in the avian stem lineage.

**Fig. 4:**
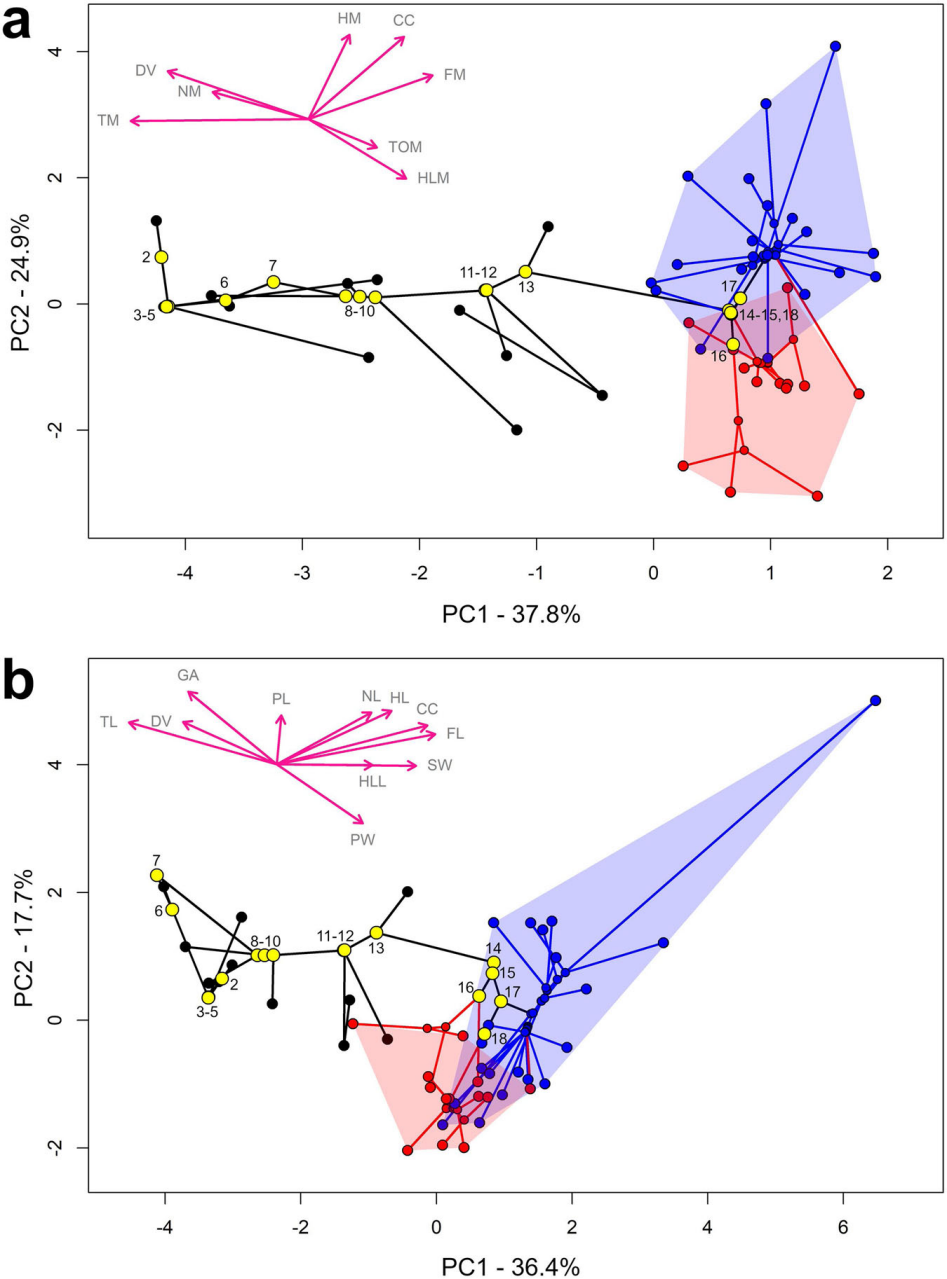
Phylomorphospace plots of PCA analysis of body segment evolution. PCA scores of individual taxa and reconstructed ancestral state nodes, showing values of relative body segment (**a**) masses and (**b**) linear dimensions in hindlimb-dominated (HLD) and forelimb-dominated (FLD) extant birds and extinct non-avian archosaurs. The larger yellow circles represent the major reconstructed nodes through avian evolution, and are numbered as follows: 2. Archosauria, 3. Dinosauria, 4. Saurischia, 5. Theropoda, 6. Neotheropoda, 7. *Dilophosaurus* + Neotetanurae, 8. Neotetanurae, 9. Coelurosauria, 10. Maniraptoriformes, 11. Pennaraptora, 12. Eumaniraptora, 13. Avialae, 14. Ornithuromorpha, 15. Neornithes, 16. Palaeognathae, 17. Neognathae, 18. Galloanserae. Blue data points/lines indicate FLD locomotor assignment, red data points/lines represent HLD locomotor assignment, and black data points/lines represent extinct non-avian archosaurs. PC loading vector abbreviations: CC cranio-caudal CoM, DV dorso-ventral CoM, HD head mass, NK neck mass, TO torso mass, TM tail mass, FM forelimb mass, HLM hind limb mass, HL head length, NL neck length, SW shoulder width, GA gleno-acetabular length, HLL hindlimb length, TL tail length, FL forelimb length, PL pelvic length, PW pelvic width. Source data are provided as a Source Data file.

In PCA analyses we recover evidence for segregation between extinct non-avian archosaurs, HLD and FLD birds in body segment mass (Fig. 4a, Supplementary Data 34) and linear parameters (Fig. 4b, Supplementary Data 33) on axes PC1 and PC2, which collectively account for 63% and 54% of the variation in the two analyses (Supplementary Data 30). In the PCA of body segment masses, PC 1 shows a strong, almost linear phylogenetic trend with scores on this axis increasing along the avian stem lineage, culminating in the highest scores in extant birds (Fig. 4a). Avialae (*Archaeopteryx*) lies outside PC1 range of extant birds, with Ornithuromorpha (*Yixianornis*) being the first node to lie within extant bird morphospace (Fig. 4a). Extant FLD and HLD birds show some segregation on PC2, with FLD birds tending towards higher scores on this axis. PC1 is most strongly correlated with torso mass, DV_CoM, tail mass and forelimb mass, while PC2 is dominated by variation in CC_CoM, hindlimb mass, tail mass and forelimb mass (Supplementary Data 32). This parameter variation is such that extinct non-avian taxa and nodes are found in areas of the morphospace with lower torso mass and forelimb mass, higher tail mass and more dorsal DV_CoM positions than extant avian taxa and nodes (Fig. 4a). FLD birds are found in areas of morphospace with more cranial CC_CoM positions, higher forelimb and head masses, and lower torso and hindlimb masses than extant HLD birds (Fig. 4a).

In the PCA of body segment linear dimensions, PC 1 also shows a strong, almost linear phylogenetic trend with scores on this axis increasing along the avian stem lineage, culminating in the highest scores in extant birds (Fig. 4b). However, unlike the analysis of segment masses (Fig. 4a), FLD and HLD birds also show relatively strong segregation on PC1, with FLD birds tending to have higher scores on this axis (Fig. 4b). Overlap on PC1 between stem avian nodes (and their associated extinct taxa) and the extant HLD bird range occurs between Eumaniraptora and Avialae (Fig. 4b), thus more basally than in segment mass parameter morphospace (Fig. 4a). FLD birds and most extinct non-avian taxa and associated nodes generally show higher scores on PC2 than HLD birds. PC1 is most strongly correlated with more cranial CC_CoM positions, increasing forelimb and GA length, and moderately correlated with most other parameters, while PC2 scores are most strongly correlated with increasing hindlimb length and shoulder width and decreasing pelvic and neck lengths (Supplementary Data 31).

There is little evidence for phylogenetic and locomotor segregation on PC3 for either body segment masses or linear dimensions (Supplementary Fig. 8). Removal of the pelican had extremely modest quantitative impact on the segment mass PCA morphospace and thus no effect on the qualitative trends noted above (Supplementary Fig. 7a, c). In the linear dimensions PCA, removal of the pelican had similarly negligible effect on PC1, but did serve to shift the qualitative distinction between locomotor groups seen in PC2 in the full data set (Fig. 4b) to PC3, where FLD birds and most extinct non-avian taxa and associated nodes are somewhat segregated from HLD birds by variations in hindlimb length, tail length and neck length (though note that CC_CoM and DV_CoM also contribute strongly to this variation with the pelican removed; Supplementary Data 31).

## Discussion

Despite fundamental expectations of mechanistic links between body shape and the mechanics of movement^1–5^ and hypotheses linking mass distribution to the evolution of avian locomotion^6–9,12^, few studies to-date have quantitatively addressed the associations between 3D body proportions, mass distribution and locomotor ecology in extant birds. Our new data suggest that FLD (predominantly volant) and HLD (predominantly terrestrial) birds exhibit significant differences in individual body segment proportions (Supplementary Figs. 10–11), in their collective multivariate body proportions (Fig. 4) and in their whole-body CoM position (Fig. 2). In our sample, HLD and FLD birds do not overlap in CoM morphospace, largely due to a more caudal CoM position in HLD taxa (Fig. 2). Categorization of any animal group into locomotor categories is to an extent an arbitrary practice, and in this case our data set includes taxa that habitually engage in both terrestrial (HLD) and aerial (FLD) locomotion to varying degrees. However, the patterns in body shape and mass distribution recovered here correlate with clear mechanical benefits in bipedal terrestrial *versus* flying locomotion and therefore shed light on adaptations and competing constraints that may have shaped ecologically-related diversity in the avian body plan. A more cranial and ventral mass distribution in FLD birds brings the CoM relatively closer to the shoulder joint and is likely to contribute to improved stability in gliding and flapping flight behaviours^28–30^. For example, a more ventral CoM relative to the centre of lift produced by the wings provides passive “pendulum” stability to the system by resisting pitch and roll^29^. Conversely, a more caudal CoM position will realise a reduction of external moments acting on hindlimb joints during bipedal terrestrial locomotion^13,16^, lowering muscle activations and reducing energy costs. This mechanistic relationship between CoM and limb mechanics likely underpins the disparate allometric patterns we recover between HLD and FLD birds in body proportions and overall CoM position: HLD birds have CoM positions increasingly closer to the hip as body size increases, whereas CoM position scales isometrically (i.e. remains relatively constant) in FLD birds. It is possible that isometric CoM scaling in FLD birds represents a modular morpho-functional constraint related to flight, and that, unlike in HLD birds, both the hindlimbs and forelimbs are under pressure to maintain locomotor performance as body size increases given the need to undertake at least some terrestrial locomotion. Given these findings it might be interesting for future studies to examine correlations between CoM and specific aspects of functional anatomy related to both flight (e.g. forelimb muscle mass, wing area) and terrestrial locomotion (e.g. hindlimb muscle mass), thereby providing more granular or continuous measures of locomotor specialisation as opposed to our discrete categorisation of birds as HLD or FLD.

This new understanding of mass distribution in extant birds challenges the long-standing dichotomy thought to exist between non-avian theropod dinosaurs and birds^6–9,12^. It has long been accepted that the dinosaurian ancestors of birds, with their long muscular tails and small forelimbs, had a CoM close to the hip, while modern birds, with their reduced tails and enlarged wings have their mass centred more cranially. Here we suggest that all non-avian theropod dinosaur taxa and avian stem nodes modelled here have CC_CoM positions within the range seen in extant HLD birds, regardless of the extant analogue and reconstruction method used to derive their skeletal to skin volume ratio (Fig. 2, Supplementary Figs. 3–4). The single exception to this is *Staurikosaurus* and the associated prediction for the early Theropoda node, where some model iterations yield a CC_CoM position slightly caudal to CoM range recovered here for HLD birds (Fig. 2, Supplementary Fig. 3), but the balance of models and overlapping 95% confidence intervals means a position within the extant HLD range is more strongly supported. Thus, rather than a qualitative phylogenetic distinction in CoM position between “dinosaurian” and “avian” conditions, we recover a locomotor-based dichotomy: HLD non-avian dinosaurs and birds have a more caudal CoM than FLD taxa irrespective their phylogenetic placement (Fig. 2). While we recover a strong cranial CoM migration across the avian stem lineage purported by previous studies^6–9,12^, we demonstrate that this migration moved across the CoM morphospace seen in extant HLD birds, culminating in a shift into FLD CoM morphospace at Avialae (*Archaeopteryx*) at the origin of powered flight (Fig. 2).

Our data also suggest that the morphological drivers of CoM evolution along the ancestral bird-line were more complex than previously suggested. Qualitative analyses have suggested that tail reduction drove cranial CoM migration in non-avian theropods^6,7^, while quantitative approaches previously recovered statistically significant correlations with enlargement of the forelimbs and reduction of the hindlimbs only; correlations to other body segments, including the tail, were not statistically significant^9^. Here, however, we recover statistically significant correlation between numerous body segment proportions and reconstructed ancestral state CoM positions (Supplementary Data 25–29). Along the avian stem-line, tail mass and length show the strongest correlations, followed by individual forelimb segments and the whole forelimb overall. However, we recover significant contributions from other previously unconsidered body proportion measures, specifically decreasing pelvic width, increasing shoulder width and GA length, and increasing torso mass. These parameters also have a strong influence on trends in PCA analyses, contributing to the segregation of non-avian dinosaurs and extant HLD and FLD birds in body proportion morphospaces (Fig. 4). Each of these changes may be mechanistically linked to trade-offs between locomotion and overall body shape change; for example, flight aerodynamics would benefit from a maximising streamlining of the torso (decreasing pelvic width and increasing GA length) whilst maximising “locomotor” muscle mass in the pectoral girdle and forelimb (increasing shoulder width and torso mass). Increasing torso mass may also be partially connected to tail reduction, with hip extensor muscle mass becoming more concentrated around the pelvis (part of the torso segment in our models).

Previous work has suggested disintegration or decoupling of forelimb and hindlimb lengths at the origin of birds, resulting in more independent control of limb development to dissociate limb lengths from body size^31^. However, we find that normalized hindlimb and forelimb lengths are very strongly correlated (raw taxon data and ancestral states) to CoM and each other, both within the ancestral bird-line and across our whole data set (Figs. 3a, b, 4a). While here we assess CoM in standardised ‘neutral’ postures rather than habitual locomotor postures, the qualitative effects of hindlimb and forelimb expansion (or reduction) on CoM will be the same in both cases given these segments will lie caudal (hindlimb) and cranial (forelimb) to the overall CoM. The correlations noted above between limb segment size and mass distribution make sense in terms of CoM constraints on basic locomotor mechanics and in the context of bird-line evolution; powered flight demands expansion of the forelimb locomotor module, which in isolation would shift the CoM cranially. Coupled, but perhaps less extreme, lengthening of the hindlimbs will have three synchronised effects that might mediate the negative effects of cranial CoM migration on function of the hindlimb locomotor module. First, longer hindlimbs will reduce the magnitude of cranial CoM migration itself as the forelimb expands. Second, longer hindlimb segments will reduce the amount of joint excursion required to place the feet under a more cranial CoM, potentially minimising the decrease in limb mechanical advantage^13,16^. Third, longer hindlimbs generally facilitate increased stride lengths and reduced energy costs in terrestrial locomotion, which in the specific context of cranial CoM migration in bird-line taxa may provide some compensation for more flexed joint postures (see below). Thus, while disparate allometric patterns may play some role in the evolution of forelimb and hindlimb lengths in bird-line archosaurs^31^, the strong integration of these locomotor modules we recover here is mechanistically consistent with mechanical demands of CoM position on their locomotion and its evolution (Figs. 2–4).

Our results may provide new resolution on the emergence of the “fully” crouched bipedalism seen in extant birds (Figs. 2, 3). Some studies have suggested postural change began in early Tetanurae^6,7^ or later early Eumaniraptorans^9,17,20^, while others have suggested that the “fully” crouched condition seen in extant birds arose rapidly around the base of Avialae^8^ or alternatively more gradually well within Neornithes^9^. Here, we recover a clear ventral shift in CoM in early Maniraptoriformes (ornithomimids, caenagnathids, dromaeosaurids) that brings these taxa into extant HLD bird CoM morphospace (Fig. 2). This ventral shift in CoM is correlated with an increase in hindlimb length (Fig. 3a, Supplementary Fig. 13) and mass (Supplementary Fig. 14) and occurs concomitantly with a reduction of tail-based hip extensor musculature^9^ and some alterations to key pelvic limb muscle moment arms^17,18^, providing support for acquisition of more crouched postures in early Maniraptoriformes. However, limb proportions are also a key determinant of posture^11^ and our data suggests that femur-metatarsal length proportions seen in extant birds did not evolve until Neornithes or even Palaeognathae (Fig. 3c, d). The qualitative reversal we recover in the relationship between relative femur length and CC_CoM is also likely highly critical to the evolution of flexed bipedalism (Fig. 3e). Elongation of the femur between Neotetanurae and Avialae may have evolved to minimise the degree of hip flexion as the CoM migrated cranially (Fig. 3e), allowing the knee to remain cranial to the CoM around midstance^13–16^, thereby potentially helping to maintain ancestral hip-driven locomotion to some degree. Subsequent shortening of the femur and maintenance of a relatively cranial CC_CoM position at Ornithuromorpha realises a reversal in this modular relationship and a shift into the morphospace occupied by extant FLD birds (Fig. 3e). This modular reversal provides support for a substantial shift in limb posture at Ornithuromorpha, with the highly crouched system seen in extant birds potentially evolving here or in the earliest Neornithes.

These evolutionary patterns in mass distribution and limb proportions therefore suggest that the “fully” crouched bipedalism seen in modern birds evolved after powered flight and its associated cranio-dorsal CoM position, rather than as an exaptation to flight and its associated body shape (Fig. 2). Indeed, Avialae is the first node to lie exclusively within the more cranial CoM morphospace recovered for extant FLD taxa, while reconstructed ancestral states for Neornithes, Neognathae and Galloanserae are located firmly within forelimb-dominated morphospace. Contrary to previous hypotheses^32^, this suggests that ancestral Neornithes were well-adapted for powered flight and that CoM positions more mechanically advantageous to terrestrial locomotion arose through major reversals in ratites and Galliformes.

As with most palaeontological studies, our analyses of evolutionary patterns are limited by the data available in the fossil record. For example, controlling for ontogenetic changes in body proportions is challenging given the availability of near-complete fossil specimens. Previous volumetric work on dinosaur body proportions has recovered evidence that CoM may be more cranial in larger, more mature specimens of *Tyrannosaurus*, owing to the torso becoming longer and heavier while the limbs become proportionately shorter and lighter^33^. Similarly, CoM positions for smaller, juvenile specimens of ratites derived from CT body volumes yielded slightly more ventral CoM positions to the larger, adult specimens in this study, again (as in *Tyrannosaurus*^33^) due to their proportionally longer legs^34^. Here, we modelled the Berlin specimen of *Archaeopteryx*, which, like all known near-complete specimens, is considered as juvenile^35^. Linear bone and body segment proportions are relatively similar in this specimen to the largest near-complete Solnhofen individual (generally around 25% larger^36^), but it is possible that the CoM position of fully mature *Archaeopteryx* could differ slightly to the values presented here. However, based on the findings noted above^33,34^, it might be predicted that adult CoM positions would be slightly more cranial and particularly dorsal to the skeletally immature Berlin specimen, which would strengthen rather than weaken its placement within extant FLD CoM morphospace (Fig. 2).

Although our sample of fossil taxa draws on representatives of most major non-avian theropod groups spanning the bird-line, other groups key to understanding the origin of Avialae and the evolution of flight (e.g. *Rahonavis, Scansoriopteryx)* are yet, to our knowledge, to be analysed by volumetric modelling approaches, particularly where specimens are unrepresented by near-complete three-dimensionally preserved specimens. Unusual morphologies and the limitations of fossil preservation, and particularly the challenges of reconstructing biomechanical performance from fossilised hard tissue alone^12,13,16,19,36–41^, mean that the locomotor capabilities of these taxa remain somewhat controversial^42,43^, although recent description and analysis of paravians with preserved muscle and body segment outlines have provided key insights into early flight evolution^44^. Given their skeletal proportions and likely phylogenetic positions, analyses of mass distribution in these groups potentially could refine or add a higher degree of complexity to the trajectory of CoM evolution recovered here between Maniraptoriformes and Avialae (Fig. 2b), including pushing the cranial shift we recover at Avialae more baseward (Fig. 2). Furthermore, our sample size of extinct non-avian theropods also limits our ability to examine the relationship between body proportions, CoM and overall body size along the bird-line. Previous comparisons of CoM within theropod groups that evolved very large body size have provided no evidence for differences between ‘medium’ and large-bodied taxa^45^, but so far these studies have not considered the full size ranges present in these lineages. While we recover little correlation between body mass and CoM positions in our bird-line sample (Supplementary Data 20–21), the ventral CoM positions in coelurosaurs and the cranial shift in CoM at Avialae (Fig. 2) do coincide with smaller body sizes in our modelled taxa. Analysis of large data sets of limb bone measurements has suggested that small body size was as a key biological factor in phylogenetic and ecological diversification on the evolutionary line leading to birds^46^. Understanding how body shape and mass distribution fit into patterns of size evolution in future studies may yield important insights into bird evolution, as well as intrinsic constraints on body proportions and locomotion.

## Methods

### Body proportions and CoM in extant birds

Thirty-three skeletal and skin volume models of extant birds were generated using our previously well-validated methodology^47–49^. These birds provide broad coverage of the phylogenetic, locomotor and body shape diversity seen in extant birds (Supplementary Data 1). 3D digital skeletons and closed skin volumes were extracted from CT and µCT scans of whole cadavers using either Mimics (version 23) or Avizo (version 9) and split into functional body segments. Models were imported into Autodesk Maya software (Versions 2016 and 2021), and both skeletons and skin volumes were rotated into a standardised neutral/reference posture through rotation of segments about joint centres between adjacent segments (Fig. 1b, Supplementary Fig. 1). Standardisation of posture is crucial for meaningful comparisons of CoM and assessing correlations between mass distribution and body proportions^4,9,37,47–49^. The posture used here was chosen on the basis that it represented one that could be repeatably and objectively applied to all taxa. One obvious difference between the chosen standardised posture (Fig. 1b, Supplementary Fig. 1) and the more ‘habitual’ postures of at least most extant birds lies in the neck, which is fully extended in our models and but often posed in a “s-shape” by live birds. With little to no quantitative data on most frequently used neck postures in birds we choose an extended posture because it could be repeatably and objectively produced in all species. Variation in cervical counts across birds and the high levels of redundancy in posture across the large number of cervical joints meant any deviation from such a posture would be highly subjective and difficult to implement objectively across birds (and may ultimately not reflect habitual postures anyway). However, to demonstrate the effect of applying a qualitatively defined s-shaped neck posture on CoM in our extant birds we carried out a sensitivity test (Supplementary Fig. 2). In this sensitivity test, we rearticulated the necks of 10 birds into what we subjectively felt was a generic ‘s-shaped’ avian neck posture. The 10 species were chosen specifically because they incrementally span the range of CoM positions across the data set, allowing observation of how rearticulation of the neck impacts the spread of data. As would be expected, switching to an approximately s-shaped neck moved the CoM of all birds caudally and dorsally. This effect was slightly greater in birds with large necks and heads like the pelican, but such birds have the most cranial CoM positions and so the result would be a dilution of the cranial extreme of the FLD group CoM range (Fig. 2a). However, overall neck posture is unlikely to influence the qualitative finding of more cranial CoM positions in FLD versus HLD birds, which is perhaps not surprising given that we recovered no statistically differences between FLD and HLD birds in neck length and mass (Supplementary Data 18–19).

Once articulated in the neutral posture, body segment lengths were calculated as the distance between joint centres and normalised by body mass^0.33^ for all comparative statistical analyses (see below). Three anatomical landmarks were placed on the sternum and the distances between them calculated to represent the approximate depth and length of the sternum (Supplementary Fig. 1). Mass properties data were calculated for each body segment skin volume using a density of 1000 kgm^−3^, with the exception of the neck (800 kgm^−3^) and torso (850 kgm^−3^) segments, which are given lower densities to account for respiratory structures like lungs and air sacs^48,50^. These standardized values were chosen in the absence of accurate species or larger clade-specific values for extant archosaurs. We tested the impact of these assumed values for extant and extinct taxa (see below) by re-running our analyses in two other segment density scenarios. First, we set all segments set to a density of 1000 kgm^−3^ to examine the pattern of body shape evolution given purely by segment volume and in the absence of any subjective investigator choice for segment density. Previous evaluations of volumetric models have independently concluded that use homogeneous density resulted in very similar CoM estimates to more realistic heterogeneous density values in birds^48,49^. Second, we produced an iteration of our analysis where all individual taxa had heterogeneous segment densities (to account for respiratory structures like lungs and air sacs^45,47^), but these densities varied across major groups. Specifically, we varied neck and torso densities between extinct non-avian sauropsids (neck 850 kg m^−3^, torso 900 kg m^−3^, other segments 1000 kg m^−3^), HLD (neck 825 kg m^−3^, torso 875 kg m^−3^, other segments 1000 kg m^−3^) and FLD birds (neck 800 kg m^−3^, torso 850 kg m^−3^, other segments 1000 kg m^−3^) to examine how potential (but untested) density reduction due to increased skeletal pneumaticity along the bird-line and in volant taxa^51^ might impact on CoM trends. Both these additional density iterations showed extremely minor quantitative differences to the original standardised heterogeneous density iteration in our main analyses (Supplementary Figs. 3–4). The CoMs for all individual segments were used to calculate whole-body CoM by multiplying the segment masses by the Cartesian coordinates of their CoMs and dividing the sum of these by the total body mass. In our statistical analyses (see below), segment mass was used to evaluate the pure “size” effect of individual segments on overall CoM, and where necessary this parameter was normalised by dividing by total body mass.

We sought to examine the relationship between mass distribution, body proportions and locomotor ecology at the coarsest level by categorising extant birds as either hindlimb-dominated (HLD, predominantly terrestrial) or forelimb-dominated (FDL, predominantly volant) in terms of locomotion. This system follows the general scheme outlined by Heers and Dial^52^ based on a combination of habitual locomotor strategies and relative performance in hindlimb-dominated activities on the ground versus forelimb-dominated aerial locomotion^5,52–56^. We chose this simple scheme specifically because our focus here lies in the evolutionary transition between terrestrial and volant locomotor modes during the evolution of birds. While further or more complex locomotor sub-categorisation of birds (e. g., hindlimb-assisted sub-aqueous diving) may be warranted in other contexts, we felt such schemes were not directly relevant to the evolutionary and ecological transitions we seek to analyse here (Figs. 1–4). Where species change locomotor habits and/performance during ontogeny, the adult condition was used to categorise birds. For example, mallards exhibit a relative increase in wing performance and decrease in hindlimb performance during ontogeny, which is linked to their shift towards greater volant locomotor ecology in adulthood^52–54^.

Phylogenetic generalised least squares (pGLS) regression^57^ was used to model the relationships between CoM, locomotor mode, body size and individual body segment properties in birds in a phylogenetic framework in R using the nlme v. 3.1–144 and ape v. 5.3 packages (Supplementary Code 1). Models were compared based upon rankings of AICc scores. Differences in the relative size of body segments were tested for using phylogenetic ANOVAs (pANOVAs) in the R package RRPP v. 0.6.1^58^ (Supplementary Code 2). These analyses of extant birds used a distribution of supertree topologies from previous analyses^59^. We re-ran these analyses twice to investigate the impact of ‘outlier’ taxa on the findings, first removing ratites (i.e., by far the largest birds, and among those with the most caudal and ventral CoM positions) and then separately removing the pelican (which has the most extreme cranial CoM position).

### The evolution of body proportions in bird-line archosaurs

To assess trends in the evolution of body proportions and locomotion during the evolution of birds, we generated measured linear body segment lengths and estimated skin volume data based on existing 3D digitized fossil skeletons of 14 taxa^9,24,25,56,60^ (Fig. 1). Taxa modelled were *Batrachotomus, Heterodontosaurus*, *Staurikosaurus, Plateosaurus*, *Coelophysis*, *Dilophosaurus, Allosaurus*, *Tyrannosaurus*, *Struthiomimus*, *Anzu, Microraptor, Velociraptor, Archaeopteryx* and *Yixianornis*. These digital skeletal models come from Allen et al.^9^, except *Allosaurus*^25^ (MOR693) and *Tyrannosaurus*^24^ (formerly BHI3033) which were used instead because of their larger size and/or better completeness, and *Batrachotomus* which comes from Bishop et al.^60^. The models of *Marasuchus* and *Pengornis* from Allen et al.^9^ were not complete enough for the method of volumetric reconstruction used herein (see below) and were therefore not used. The skeletal models of *Anzu* and *Archaeopteryx* we re-scaled isometrically to amend the scaling in Allen et al.^9^, but this had no effect on the model’s segment proportions and thus would not change the size-normalised CoM estimates in this previous study (Supplementary Tables 4–7).

Digital skeletal models were articulated in the same standardised reference postures as the birds and linear body segment lengths calculated as the distances between joint centres. To reconstruct body segment skin volumes, and subsequently whole-body mass properties, we used the minimum convex hull (MCH) approach^4,60–64^ (Fig. 1b). The MCH (enclosed volume) around each segment was calculated using the Matlab (www.mathworks.com) qhull algorithm. This mathematical approach of tightly fitting three-dimensional convex polygons to each body segment minimizes subjectivity in body volume reconstruction. In addition, the extent of an object’s MCH is dictated solely by its geometric extremes, which minimizes impact of reconstructed (i.e. missing) skeletal components in fossil skeletons^4,62^. The volumetric properties (volume, CoM position) of each body segment’s minimum convex hull was calculated in MeshLab 2021 (www.meshlab.net). The MCHs are then expanded around fossil skeletons according to scaling relationships between MCHs and mass properties measured in living animals^4,60–63^. However, previous studies have used whole-body scaling factors, which limit studies of fossils to homogenous expansion of all body segments, which is unlikely to be realistic. Here we overcome this issue by generating body segment-specific MCH expansion factors for living archosaurs using our 33 avian volumetric models and an additional 17 models of extant lepidosaurs and crocodylians (Fig. 1; Supplementary Data 2). The lepidosaur and crocodylian models were generated using the same approaches described for the avian skeletal and skin volume models above (Fig. 1). The relationship between actual skin volume and the MCH bone volume of each body segment was examined using pGLS and ordinary least squares regression in R using the nlme v. 3.1–144 and ape v. 5.3 packages (Supplementary Code 3). As above, the phylogenetic relationships of extant birds used^59^, while the topologies of trees including extant lepidosaurs and crocodylians were derived from timetree.org.

Minimum convex hulls for each body segment in the non-avian theropod models were expanded in four separate model iterations based on our extant data, using the (1) all extant taxa equations (i.e. 33 avian and 17 non-avian sauropsids, Supplementary Data 3), (2) avian-only equations (Supplementary Data 4), (3) non-avian sauropsid-only equations from the regression models noted above (Supplementary Data 5) and (4) the raw convex hull:skin expansion factor averaged over all 50 extant taxa (Supplementary Data 6). The allometric equation iterations (iterations 1–3) inherently considered size-effects in the relationship between MCH and skin volume volumes in extant taxa, which may be predictively and biologically advantageous when extinct taxa fall within the body size range of the taxa sample upon which those equations are based. However, our non-avian theropod data set included large-bodied taxa that surely had body masses of one order of magnitude greater than any extant archosaur. Application of predictive relationships with negative or positive allometry seen in individual body segments in extant taxa to these large-bodied non-avian theropods may therefore potentially lead to erroneously small or large volumes in model iterations 1–3. By using the average expansion factor values, iteration 4 minimized such allometric effects and we therefore used this model iteration in statistical assessment of body shape morphospace evolution (see below), but we present all model iterations graphically to qualitatively constrain our interpretations of CoM evolution in non-avian theropod dinosaurs relative to extant birds (Fig. 2, Supplementary Figs. 3–4, 14), and to demonstrate that our qualitative conclusions are not affected by the choice of extant analogue/homologue and/or reconstruction method chosen. Within each model iteration, overall body mass was calculated as the sum of all expanded body segment masses and overall whole-body CoM was calculated by multiplying the segment masses by the Cartesian coordinates of their own CoM and dividing the sum of these by the total body mass as in previous studies^24,25^. The three density model iterations described above were applied to each of these four volume model iterations, yielding 12 model iterations per extinct taxon (Supplementary Figs. 3–4).

We also conducted tests to examine the predictive capability of convex hull approach and how potential limitations of the method may impact CoM predictions. First, we applied our “all taxa” and “bird-only” predictive convex hull:skin volume expansion ratios and allometric equations to our extant bird data set to examine (in)accuracy in predicted CoM positions relative to our skin volume CoM models. Quantitative inaccuracy was relatively low in all taxa and all four model iterations (Supplementary Fig. 9), with the exception of the HLD birds with the longest hindlimbs and FLD birds with particularly large necks and heads in the ‘all taxa’ hull:skin expansion factor model iteration (iteration 4 above) where larger quantitative error was observed (Supplementary Fig. 9a). However, in all model iterations the qualitative differences between phylogenetic and locomotor groups recovered in the main analysis (Fig. 2a) were preserved. Second, we examined the impact of simplified convex hull shape (versus the real skin volume “outline”) on CoM predictions by comparing skin volume values (Fig. 2) from four extant taxa of varied body shape and phylogenetic affinity to values generated by expanding body segment convex hulls to the same skin volume values. The impacts on segment and particularly whole-body CoM values were extremely small (Supplementary Fig. 10, Supplementary Tables 1–2), supporting the use of abstract shapes like convex hulls for CoM estimation in fossil material.

For our phylogenetic comparative analyses, we constructed an informal supertree of birds and non-avian theropods, bounded by successive outgroups (*n* = 50, see Supplementary Table 3 for details). Time-scaling was undertaken in Paleotree v.3.3.2561^65^, while ancestral state estimation and phylomorphospaces were generated using *FastAnc*, *phylomorphospace*, and *Phyl.PCA* functions of Phytools v. 1.0–162^66^ and PCA analyses performed using the *PCA* function within FactoMineR^67^ (Supplementary Code 4). To examine the relationship between individual body segment parameters and CoM positions in fossil taxa along the lineage to birds we used Spearman ranks correlations on both raw taxon and ancestral state node values.

### Reporting summary

Further information on research design is available in the Nature Portfolio Reporting Summary linked to this article.

## Supplementary information

Supplementary Information

Description of Additional Supplementary Files

Supplementary Data 1-34

Supplementary Code 1-4

Reporting Summary

## Source data

Source Data

## Data Availability

3D models and numerical input data into statistical analyses and associated code are available at 10.17638/datacat.liverpool.ac.uk/2164. Previously published models are available at: http://datacat.liverpool.ac.uk/310, 10.5061/dryad.hh74n and https://osf.io/6zamj. Source data are provided with this paper.

## Code availability

All scripts and input data required to repeat the statistical analyses are available in the Supplementary Code files and at 10.17638/datacat.liverpool.ac.uk/2164.
