# Supplementary material for: Decoupling body shape and mass distribution in birds and their dinosaurian ancestors
Source: Nat Commun. 2023 Mar 22;14:1575. doi: 10.1038/s41467-023-37317-y (PMC10033513; doi:10.1038/s41467-023-37317-y)
Supplement: Supplementary file 1 — Supplementary Information [file 41467_2023_37317_MOESM1_ESM.pdf]

Supplementary Information for

Decoupling body shape and mass distribution in birds and their dinosaurian ancestors

Sophie Macaulay, Tatjana Hoehfurtner, Samuel R. R. Cross, Ryan D. Marek, John R. Hutchinson, Emma R. Schachner, Alice E. Maher & Karl T. Bates*.

*Correspondence to: k.t.bates@liverpool.ac.uk.

For Supplementary Data 1-34 see SupplementaryData.xlsx

This file contains Supplementary Figures 1-15 and results, additional validation and data tests and methodological information (Supplementary Tables 1-7).

Supplementary Notes

Correlations between CoM and body segment proportions in extant birds – raw taxon values. We recover numerous statistically significant relationships between whole-body CoM positions and individual segment lengths and masses (Supplementary Data 14-17). In terms of segment lengths, the strongest associations are recovered between more caudal CoM positions and decreasing skull, forearm, overall forelimb, humeral, gleno-acetabular (GA) distance and neck lengths (Supplementary Data 14). Only head mass shows a statistically significant correlation with CC_CoM (Supplementary Data 16). For DV_CoM, more dorsal CoM positions are significantly correlated with decreasing lengths of individual hindlimb segments and overall hindlimb length, as well as decreasing GA and pelvic length (Supplementary Data 15). In terms of segment masses, the strongest associations with a more dorsal CoM position are decreasing shank, tarsometatarsal and overall hindlimb mass, and increasing hand segment mass (Supplementary Data 17).

CoM evolution and body segment correlations – raw taxon values. Extant non-avian sauropsids have a more dorsal CoM position than bird-line taxa, consistent with their greater axial:limb segment volumes (Fig. 2a). Non-avian dinosaur taxa had DV_CoM positions intermediate between extant non-avian sauropsids and birds until the evolution of maniraptoriform non-avian theropods, who plot more ventrally within the morphospace occupied by extant HLD birds (Fig. 2a). All non-avian dinosaurs have CC_CoM positions from all model iterations within the range recovered for extant HLD birds, except *Staurikosaurus*, which has one model iteration with a more caudal CC_CoM position, one model iteration within the range seen in HLD birds, and two that fall almost exactly on the caudal extreme of the HLD range when heterogeneous density is used (Fig. 2a, Supplementary Figure 2a). When homogeneous density is used, all four model iterations fall within the CC_CoM range of extant HLD birds (Supplementary Figure 2b). *Archaeopteryx* and *Yixianornis* are recovered with CoM positions within the morphospace occupied by extant FLD

birds, regardless of the extant analogue and reconstruction method used to derive their skeleton:skin volume (Fig. 2a). Applying our new all extant taxa and bird-only expansion models to convex hull reconstructions of birds (Supplementary Figure 4) preserves the qualitative differences in CoM position recovered by skin volume models (Fig. 2) and relationships in CoM between extant and extinct taxa.

Spearman's rank tests revealed statistically significant (*P* = <0.05) correlations between CC_CoM position and body mass (*Rho* = -0.56), thigh (*Rho* = -0.67), shank (*Rho* = -0.596), metatarsal (*Rho* = -0.692) and overall hindlimb mass (*Rho* = -0.705) in the non-avian sauropsid-only model iteration (Supplementary Data 20). No other segments in the other three model iterations were significantly correlated with CC_CoM, although tail mass was close to significance (*Rho* = -0.503, *P* = 0.069) in the model iteration that used the raw convex hull:skin expansion factor averaged from all extant taxa (Supplementary Data 20). Pelvic width (*Rho* = -0.6-0.675) and GA length (*Rho* = -0.622-0.684) were significantly correlated with CC_CoM in three of the four model iterations, while tail length (*Rho* = -0.644) was significantly correlated in the model iteration that used the raw convex hull:skin expansion factor averaged from all extant taxa (Supplementary Data 20). Correlations between individual body segment properties and DV_CoM were more numerous and stronger (Supplementary Data 21), with minor variation across the four fossil model iterations (Supplementary Data 21). Tail mass (*Rho* = -0.745-0.824), manus mass (*Rho* = -0.574-0.631), pes mass (*Rho* = -0.538-0.71), forelimb mass (*Rho* = -0.64-0.697), hindlimb length (*Rho* = -0.692-0.811) and forelimb length (*Rho* = -0.565-0.714) were significantly correlated with DV_CoM across all four model iterations (Supplementary Data 21). Humeral (*Rho* = -0.6-0.64) and forearm (*Rho* = -0.613-0.675) segment masses were significantly correlated with DV_CoM in all but the non-avian sauropsid-only model iteration (Supplementary Data 21). GA length (*Rho* = 0.622-0.684) and pelvic width (*Rho* = -0.6-0.675) showed the strongest signals overall, being recovered with significant correlations in three of the four model iterations (Supplementary Data 21). Thigh (*Rho* =

-0.538), shank (*Rho* = -0.552), metatarsal (*Rho* = -0.692) and overall hindlimb mass (*Rho* = -0.679) were significantly correlated with DV_CoM in the model iteration that used the non-avian sauropsid-only MCH expansion equations, and tail length (*Rho* = -0.644) was significantly correlated in the fossil model iteration that used the raw convex hull:skin expansion factor averaged from all extant taxa (Supplementary Data 21).

We also recover statistically significant positive correlations between hindlimb and forelimb lengths when all taxa are analysed (*rho* = 0.53, Supplementary Data 29), and particularly when only ancestral bird-line taxa are analysed (*rho* = 0.87, Supplementary Data 29).

Supplementary Methods

Assessing error in whole-body CoM position from body segment shape simplification in convex hulls. The skeletal hull for any given segment is not the same shape as the corresponding skin outline, and therefore will not necessarily have the same CoM. We sought to investigate the magnitude of these differences in a segment context (Supplementary Table 1), and to examine what effect they have on whole body CoM position (Supplementary Figure 10, Supplementary Table 2) using four extant specimens (rhea, buzzard, alligator and iguana), representing four different body plans (Supplementary Figure 10) from our data set.

At the level of individual body segments, absolute differences in segment CoM values for skin versus convex hull volumes ranged between 0-49mm across all specimens, or 0-20mm excluding the large-bodied rhea (Supplementary Table 1). The reptiles were minimally affected, with a maximum error of 6.6mm for the tail segment in the alligator (Supplementary Table 1). Certain segments of the birds were more affected, mostly the tapered limb segments (e.g. buzzard upper arm, error = 20mm; Supplementary Table 1).

To quantify the effects of convex hull shape specifically on whole-body CoM, we re-calculated whole-body CoMs by replacing skin segment volume CoM positions with the segment CoMs positions given by the skeletal convex hulls. The impact on whole-body CoM was extremely small, with the difference between the two alternative whole-body CoMs limited to 0.849-10.8mm (Supplementary Table 2). In each case, these distances were small in comparison to body size (Supplementary Figure 10), supporting the use of abstract shapes like convex hulls for CoM estimation in fossil material.

Phylogenetic comparative analysis. To analyse changes in body form across Theropoda, we constructed an informal supertree based on recent morphological and molecular phylogenetic analyses. The final tree comprises avian and non-avian theropods, bounded by successive dinosaurian, pseudosuchian, and squamate outgroups. The interrelationships of Neornithes follows the molecular analysis of Jetz *et al.*[1], while the positions of non-avian theropods were taken from recent phylogenetic analyses of theropod phylogeny[2-6].

Time-scaling was undertaken using the cal3timePalaeoPhy function of Paleotree v.3.3.25[7], implemented in R Studio v.4.1.2 (https://www.rstudio.com/). Cal3 is a probabilistic method, which samples divergence times under a birth-death model[8]. This requires estimations of sampling, branching, and extinction rates, which were devised using the native functions of Paleotree. Here, the 'minMax' date treatment setting was chosen for our analysis because our occurrence data consists of plausible occurrence ranges, as opposed to absolute dates.

Occurrence data was organised as a list of first and last appearances (FADLAD), which were taken from the literature, and are presented in Supplementary Table 3. Generally speaking, FADLAD dates were binned to geologic stage (e.g. a Campanian occurrence for *Velociraptor mongoliensis* produced an FADLAD of 83.65 to 72.17 Ma). The dates for substages were taken from the latest version of the geologic timescale[9], implemented in TS Creator v.8.0 (https://timescalecreator.org/). That said, in instances where high resolution chronostratigraphic data allowed us to assign a more precise FADLAD, we have opted to use this instead (Supplementary Table 3).

This approach will necessarily underestimate the divergence dates of extant taxa, especially when compared to molecular analyses. Therefore, to overcome this issue, all extant taxa were assigned an FADLAD of 2.58 to 0 Ma (Pleistocene to present), and following time-scaling, the trees were corrected so that the nodes and branches for the extant taxa matched the original molecular estimates, and that all extant taxa had a tip age of 0 (present day). Corrected nodes (with corresponding references for molecular estimates) comprised; Neornithes plus all daughter nodes[1], crown Crocodilia[10], crown Archosauria and crown Sauropsida (both[11]). This method ensured continuity with the trees used in our extant-only analyses, and was undertaken using the scaleTree function of RRphylo v.2.6.0[12].

Ancestral states and 95% CIs were computed using the FastAnc function of Phytools v. 1.0-1[13]. The specific parameters investigated included the heterogenous CoM and body segment masses and linear parameters. In addition to the individual ancestral state estimations, we also produced a phylomorphospace for the dorsoventral and craniocaudal heterogenous CoM estimates (Fig. 2b), for hindlimb and forelimb lengths (Fig. 3a-b) and femur and metatarsal segment lengths (Fig. 3c-d). This was computed using the phylomorphospace function of Phytools, which also uses the FastAnc function to estimate the condition of internal nodes.

Rescaling *Anzu* and *Archaeopteryx* models. Re-examination of the models of *Anzu* (= "cf. *Caenagnathus*") and *Archaeopteryx* from Allen et al.[14] showed that the linear scale was incorrect. As this scale was symmetrical in 3D, that should not affect their conclusions which were based on non-dimensional results. However, for the current study we amended the input data as follows. The *Anzu* model originally was in units of inches but off-scale slightly (~3cm/inch), presumably because it was hand-modelled from measurements of the fossil specimen, so we rescaled them for our revised model to metres by multiplying by 0.0333x. The *Archaeopteryx* model, which was laser-scanned then hand-sculpted from the original scan, resulting in a "2.5D" skeleton. The scale was off published values for skeletal elements by 1.4 times, so we increased the dimensions of the skeleton accordingly for our revised model.

Supplementary Figures

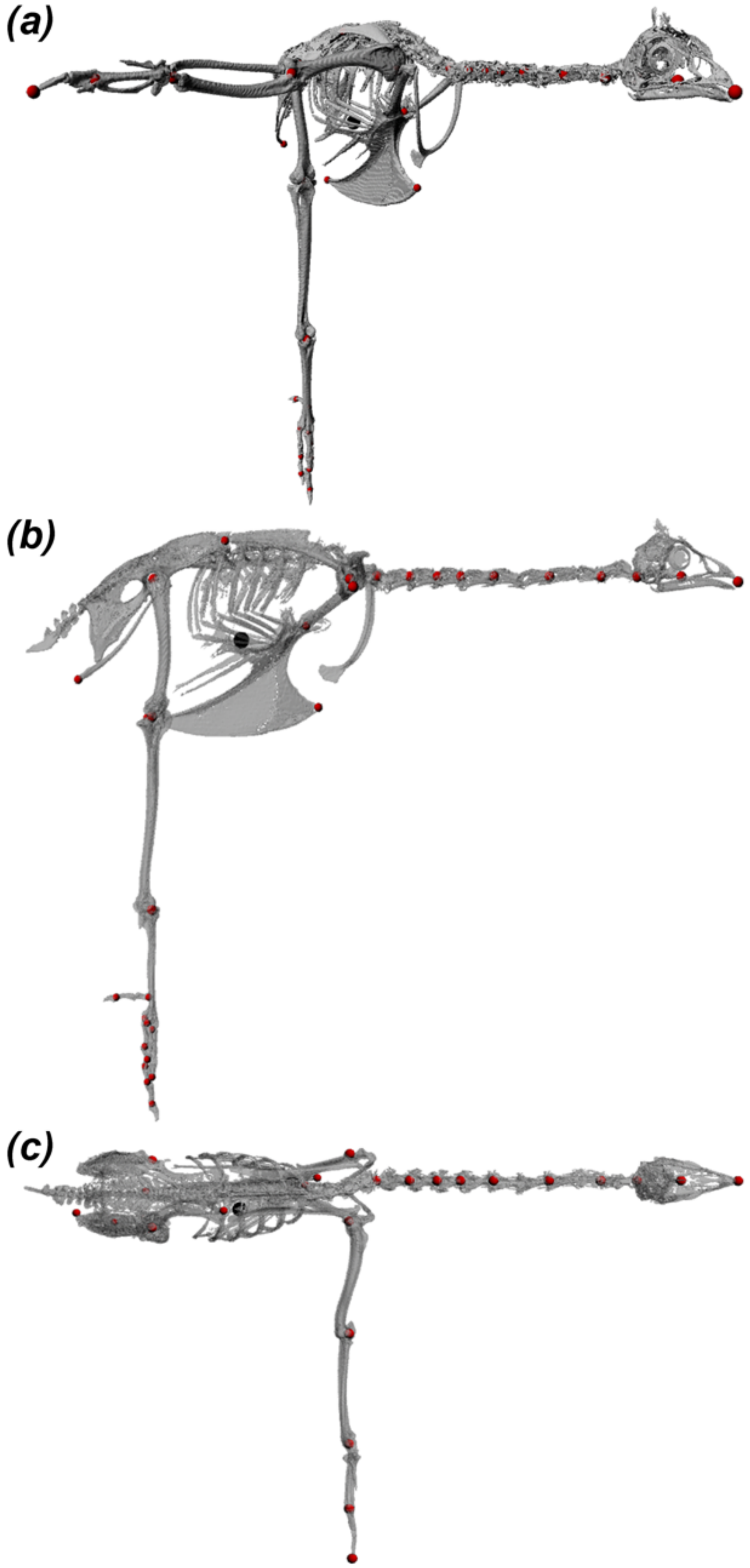

Supplementary Figure 1. Model neutral posture. Example of the joint centre and skeletal landmarks (red spheres) used to calculate linear distances for the analysis of body segment length dimensions in the extant bird data set, shown in **a.** cranio-lateral, **b.** lateral and **c.** above views. In **c**.

the centre of mass (black sphere) has been artificially displaced from the midline to aid visibility in the image.

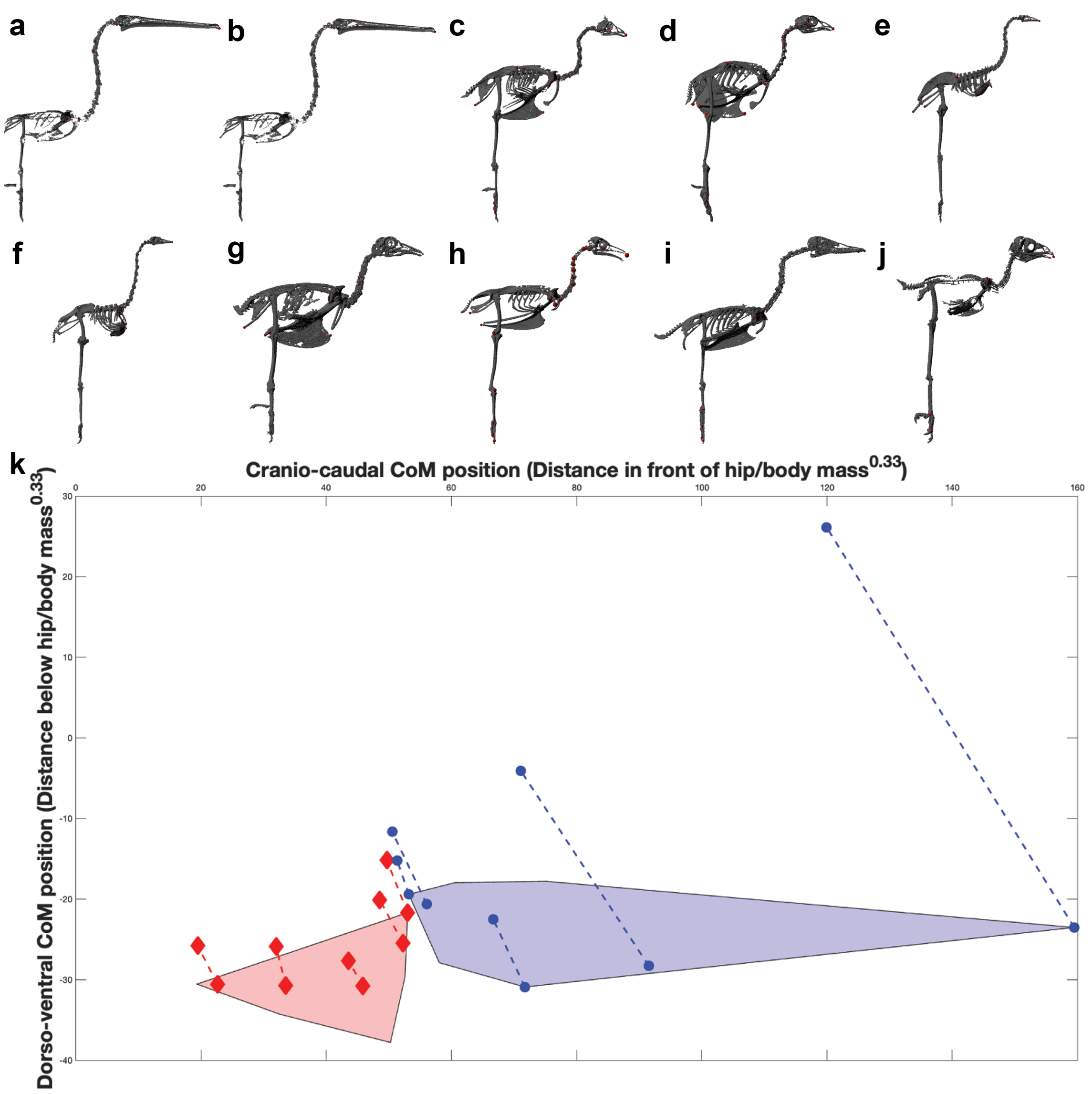

Supplementary Figure 2. CoM neck posture sensitivity analysis. Neck postures were altered and CoM recalculated in 10 species of birds to examine the effect of adopting broadly s-shaped neck postures (qualitatively/subjectively selected in the absence of quantitative data). The 10 chosen species were selected were the (a) pelican, (b) flamingo (c) guinea fowl, (d) ptarmigan, (e) emu, (f) rhea, (g). pigeon, (h) tinamou, (i) mallard and (j) buzzard. (k) The 10 species were chosen specifically because they incrementally span the range of CoM positions across the data set, allowing observation of how rearticulation of the neck impacts the spread of data, as can be seen by the distribution of each of their bottom (more cranio-ventral) data points within FLD and HLD morphospace (shaded blue and red areas). As would be expected, switching to an approximately s-shaped neck moves the CoM of all birds caudally and dorsally, as indicated by the dashed lines joining the original data points within HLD and FLD morphospaces to the more caudo-dorsal data points outside the morphospaces. This effect is slightly larger in birds with large necks and heads like the pelican, but such birds have the most cranial CoM positions and so the result would be a dilution of the cranial extreme of the FLD group CoM range (Fig. 2a). However, overall neck

posture is unlikely to influence the qualitative finding of more cranial CoM positions in FLD versus HLD birds, which is perhaps not surprising given that statistical tests recovered no statistically differences between FLD and HLD birds in neck length or mass (Supplementary Data 18-19). Source data are provided as a Source Data file.

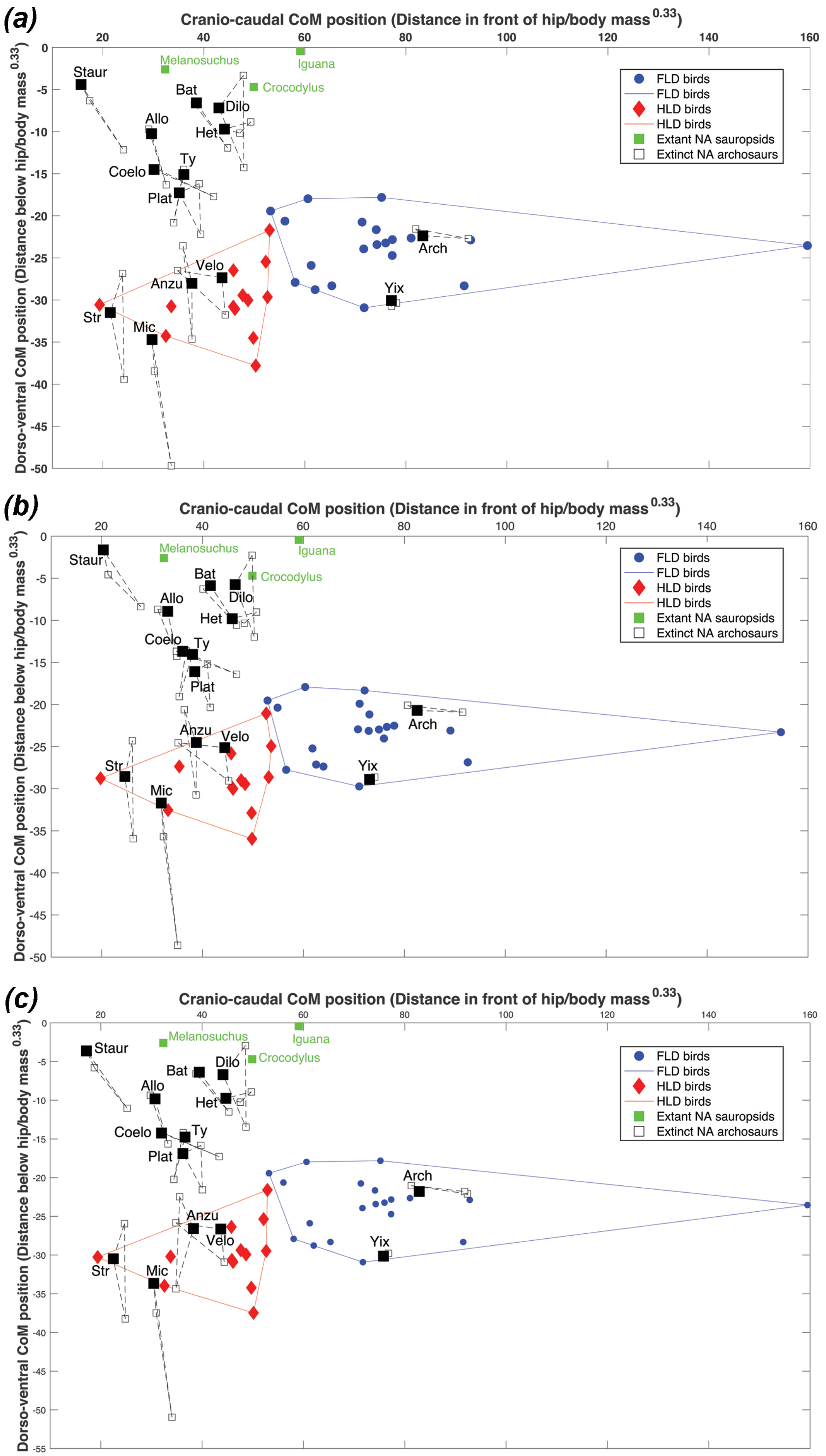

Supplementary Figure 3. CoM positions for all modelled taxa. CoM positions in non-avian saurian and birds measured from skin volumes, and predicted in bird-line fossils using new convex

hull volume expansion relationships, where the same ***(a)*** heterogeneous (neck 800kg m^{-3}, torso 850 kg m^{-3}, other segments 1000kg m^{-3}) and ***(b)*** homogeneous (all segments 1000 kg m^{-3}) density has been applied to body segment volumes (versus heterogenous density; see Fig. 2 in main text) in all taxa, and ***(c)*** where extinct non-avian sauropsids (neck 850kg m^{-3}, torso 900 kg m^{-3}, other segments 1000kg m^{-3}, HLD (neck 825kg m^{-3}, torso 875 kg m^{-3}, other segments 1000kg m^{-3}) and FLD birds (neck 800kg m^{-3}, torso 850 kg m^{-3}, other segments 1000kg m^{-3}) have been given different segment densities. These different segment density scenarios result in extremely small changes to measured and predicted CoM positions, and thus the qualitative differences between taxa are entirely preserved. Source data are provided as a Source Data file.

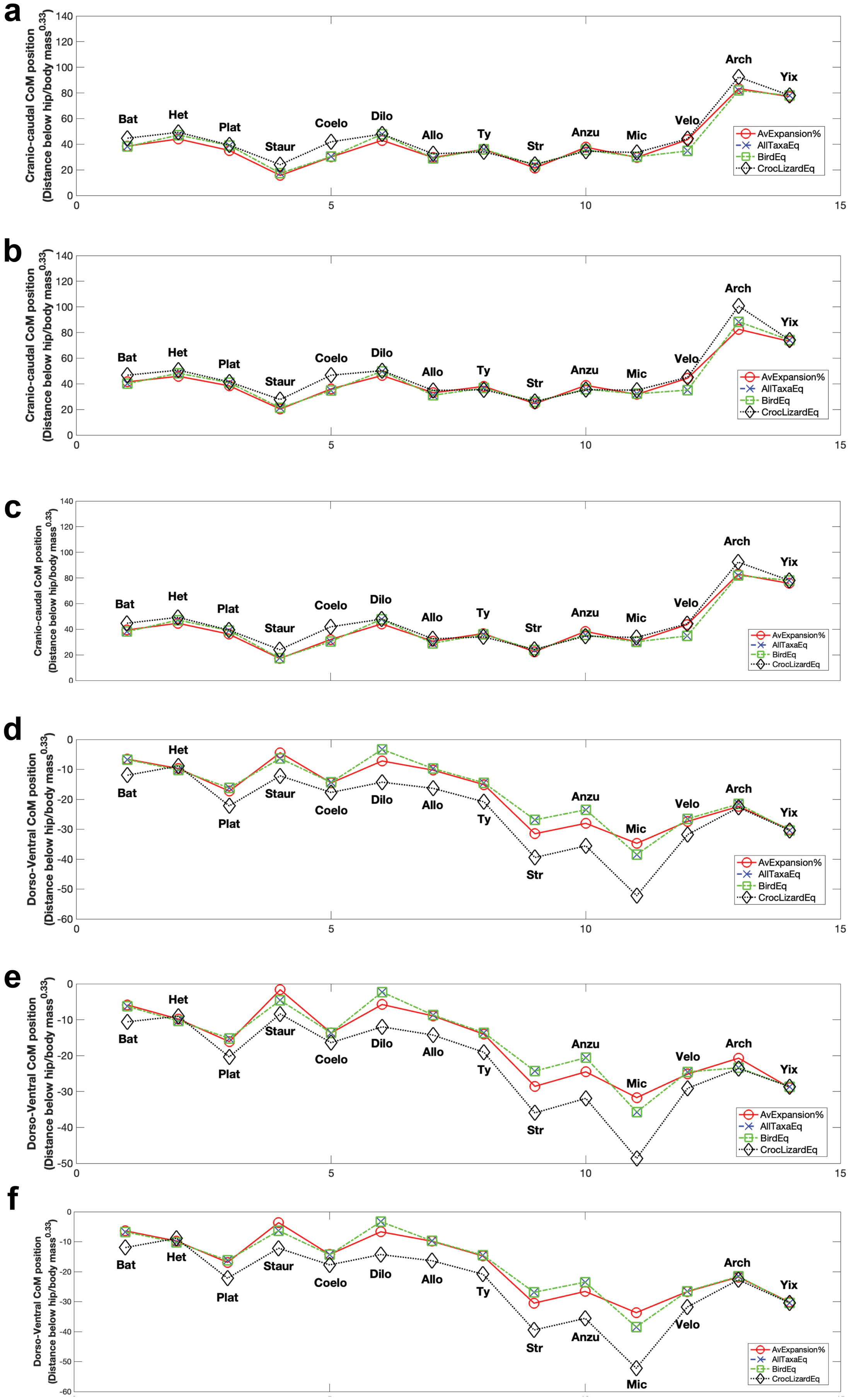

Supplementary Figure 4. Centre of mass predictions for extinct non-avian sauropsids.

Comparison of predictions for **(a-c)** cranio-caudal and dorso-ventral ***(d-f)*** CoM evolution in bird-

line fossil taxa in models where the same **(a & d)** heterogeneous (neck 800kg m^{-3}, torso 850 kg m^{-3}, other segments 1000kg m^{-3}) and **(b & e)** homogeneous (all segments 1000 kg m^{-3}) density has been applied to body segment volumes in all taxa, and (**c & f**) where extinct non-avian sauropsids (neck 850kg m^{-3}, torso 900 kg m^{-3}, other segments 1000kg m^{-3}, HLD (neck 825kg m^{-3}, torso 850 kg m^{-3}, other segments 1000kg m^{-3}) and FLD birds (neck 800kg m^{-3}, torso 850 kg m^{-3}, other segments 1000kg m^{-3}) have been given different segment densities. These different segment density scenarios result in very small quantitative differences to predicted CoM predictions, and subsequently does not alter the qualitative differences among taxa. Source data are provided as a Source Data file.

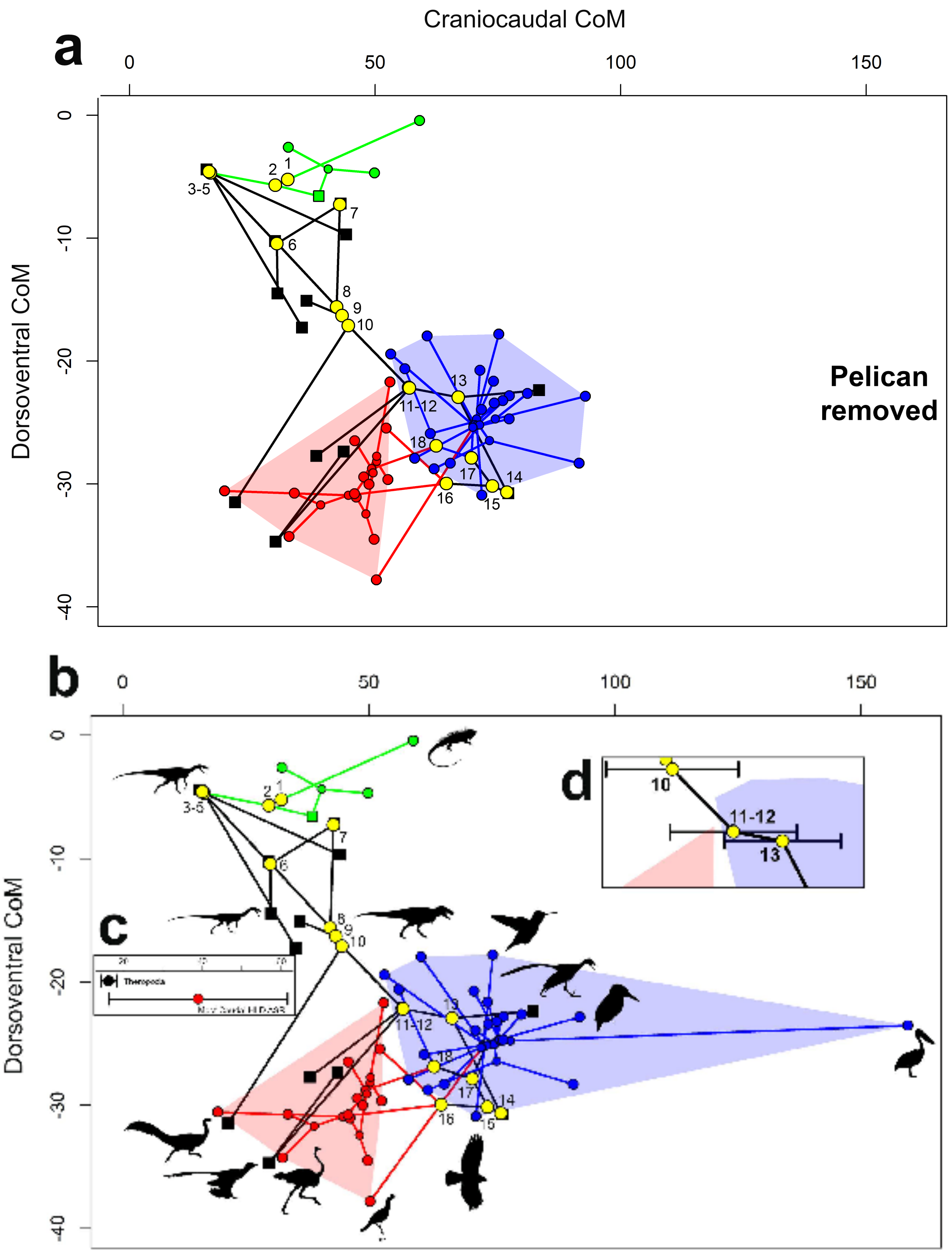

Supplementary Figure 5. Centre-of-mass evolution in bird-line archosaurs with the pelican removed. Comparison of reconstructed patterns of CoM evolution (a) with the pelican removed versus (b) the full data set (as per Fig. 2b in the main text). Both are phylomorphospace plot of the studied taxa comprising, with extinct taxa represented as squares, and extant taxa (and surviving nodes) as circles. The larger yellow circles represent the major reconstructed nodes through avian evolution, and are numbered as followed, 1. Sauropsida, 2. Archosauria, 3. Dinosauria, 4. Saurischia, 5. Theropoda, 6. Neotheropoda, 7. *Dilophosaurus* + Neotetanurae, 8. Neotetanurae, 9. Coelurosauria, 10. Maniraptoriformes, 11. Pennaraptora, 12. Eumaniraptora, 13. Avialae, 14. Ornithuromorphae, 15. Neornithes, 16. Palaeognathae, 17. Neognathae, 18. Galloanserae. **c.** Inset of the main plot (**b.**) showing the overlapping CC_CoM confident intervals of the Theropoda node and

those of the extant HLD bird node with the most caudal CoM position. **d.** Inset of the main plot. Silhouettes of *Microraptor*, *Tyrannosaurus* and the ornithomimid by Matthew Dempsey, used with permission and without modification. Silhouettes of *Coelophysis* (CC BY 3.0; https://creativecommons.org/licenses/by/3.0/) and *Herrerasaurus* (CC BY 3.0; https://creativecommons.org/licenses/by/3.0/) by Scott Hartman sourced without modification from www.phylopic.org. Source data are provided as a Source Data file.

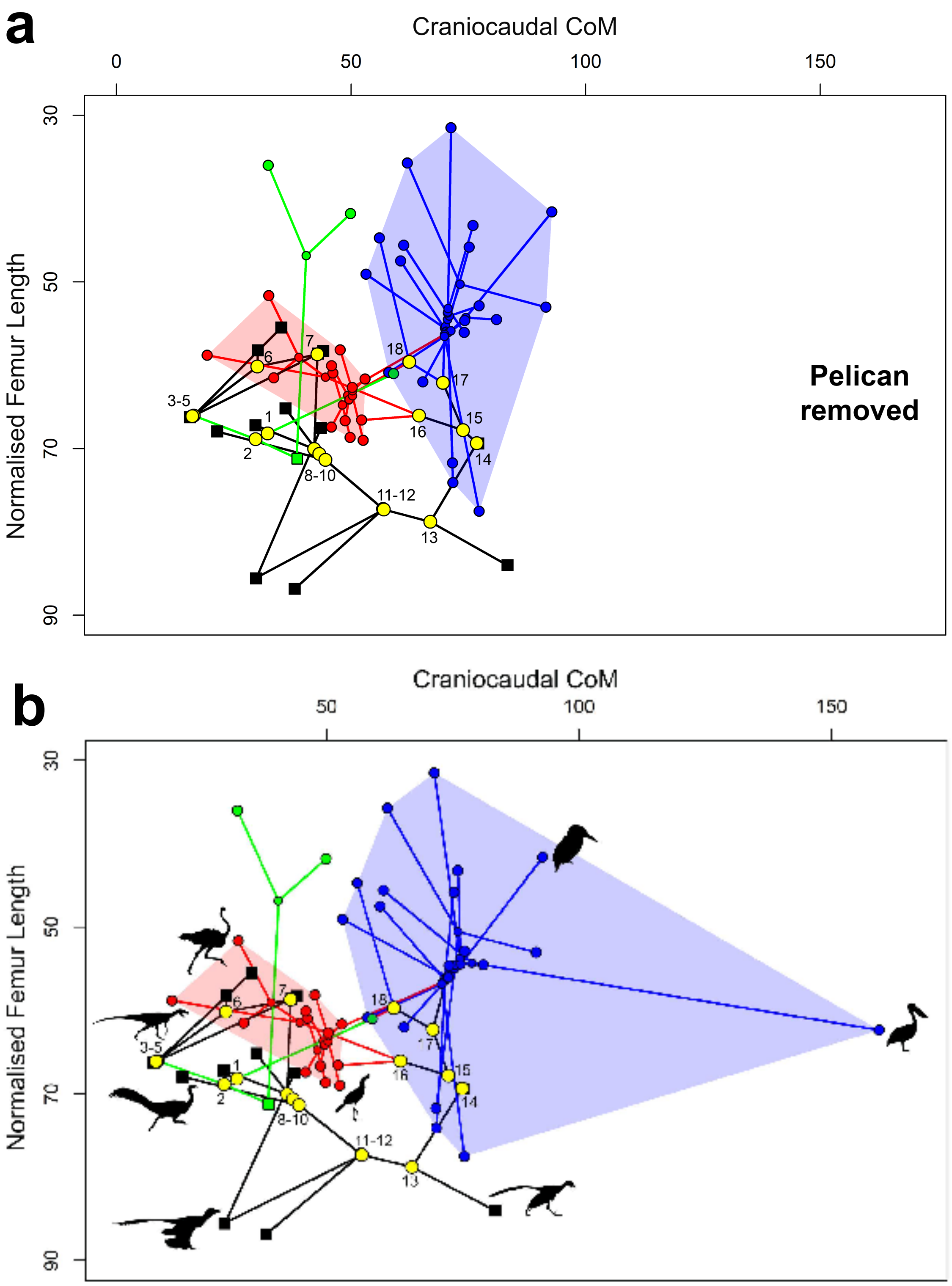

Supplementary Figure 6. Centre of evolution relative to femoral length with the pelican removed. Phylomorphospace plots of normalised cranio-caudal CoM and normalised femur length to compare the effect of (a) removing the pelican versus (b) the full data set (as per Fig. 3e in the main text). The larger yellow circles represent the major reconstructed nodes through avian evolution, and are numbered as followed, 1. Sauropsida, 2. Archosauria, 3. Dinosauria, 4. Saurischia, 5. Theropoda, 6. Neotheropoda, 7. *Dilophosaurus* + Neotetanurae, 8. Neotetanurae, 9. Coelurosauria, 10. Maniraptoriformes, 11. Pennaraptora, 12. Eumaniraptora, 13. Avialae, 14. Ornithuromorphae, 15. Neornithes, 16. Palaeognathae, 17. Neognathae, 18. Galloanserae. **e.** Silhouettes of *Microraptor*, *Tyrannosaurus* and the ornithomimid by Matthew Dempsey, used with

permission and without modification. Silhouettes of *Coelophysis* (CC BY 3.0; https://creativecommons.org/licenses/by/3.0/) and *Herrerasaurus* (CC BY 3.0; https://creativecommons.org/licenses/by/3.0/) by Scott Hartman sourced without modification from www.phylopic.org. Source data are provided as a Source Data file.

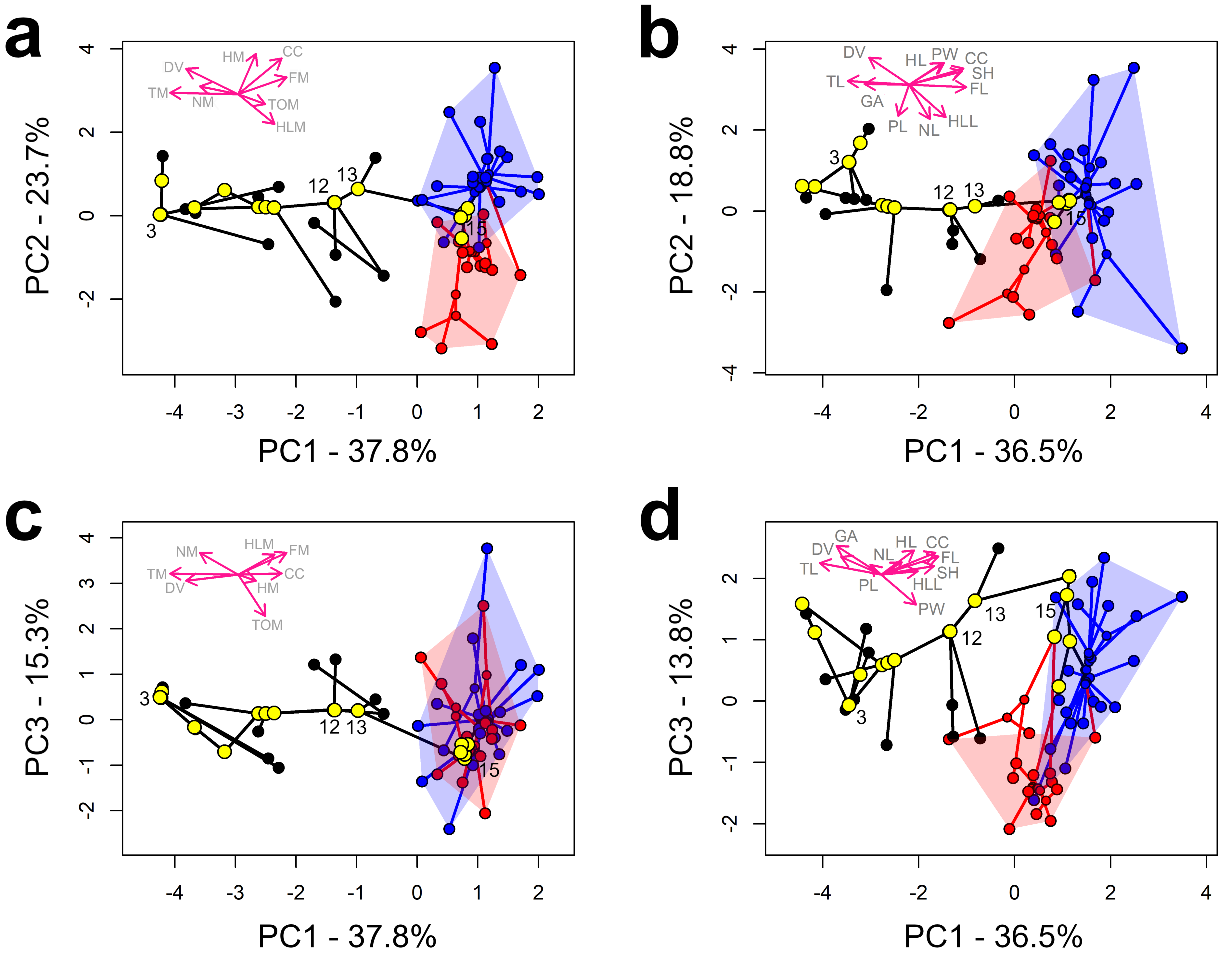

Supplementary Figure 7. Phylomorphospace plots of PCA analysis of body segment evolution with the pelican removed. PCA scores of individual taxa and reconstructed ancestral state nodes, showing values of relative body segment (**a**, **c**) masses and (**b**, **d**) linear dimensions in hindlimb-dominated (HLD) and forelimb-dominated (FLD) extant birds and extinct non-avian (NA) archosaurs, with selected nodes highlighted as follows: 3, Dinosauria, 12, Eumaniraptora, 13, Avialae, 15, Neornithes. Blue data points/lines indicate FLD locomotor assignment, red data points/lines represent HLD locomotor assignment, and black data points/lines represent extinct non-avian archosaurs. PC loading vector abbreviations: CC, cranio-caudal CoM; DV, dorso-ventral CoM, HD, head mass; NK, neck mass; TO, torso mass; TM, tail mass; FM, forelimb mass; HLM, hind limb mass; HL, head length; NL, neck length; SW, shoulder width; GA, gleno-acetabular length; HLL, hindlimb length; TL, tail length, FL, forelimb length; PL, pelvic length; PW, pelvic width. Source data are provided as a Source Data file.

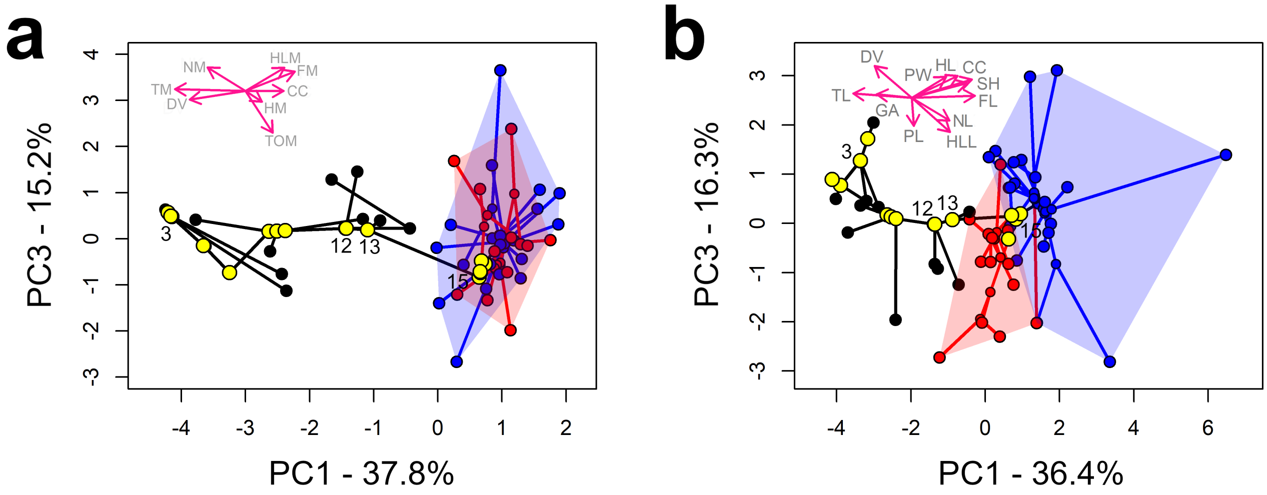

Supplementary Figure 8. Phylomorphospace plots of PCA analysis of body segment evolution showing variation on PC3. PC1 versus PC3 scores of individual taxa and reconstructed ancestral state nodes, showing values of relative body segment (**a**) masses and (**b**) linear dimensions in hindlimb-dominated (HLD) and forelimb-dominated (FLD) extant birds and extinct non-avian archosaurs. The larger yellow circles represent the major reconstructed nodes through avian evolution, with selected nodes highlighted as follows: 3, Dinosauria, 12, Eumaniraptora, 13, Avialae, 15, Neornithes. Blue data points/lines indicate FLD locomotor assignment, red data points/lines represent HLD locomotor assignment, and black data points/lines represent extinct non-avian archosaurs. PC loading vector abbreviations: CC, cranio-caudal CoM; DV, dorso-ventral CoM, HD, head mass; NK, neck mass; TO, torso mass; TM, tail mass; FM, forelimb mass; HLM, hind limb mass; HL, head length; NL, neck length; SW, shoulder width; GA, gleno-acetabular length; HLL, hindlimb length; TL, tail length, FL, forelimb length; PL, pelvic length; PW, pelvic width. Source data are provided as a Source Data file.

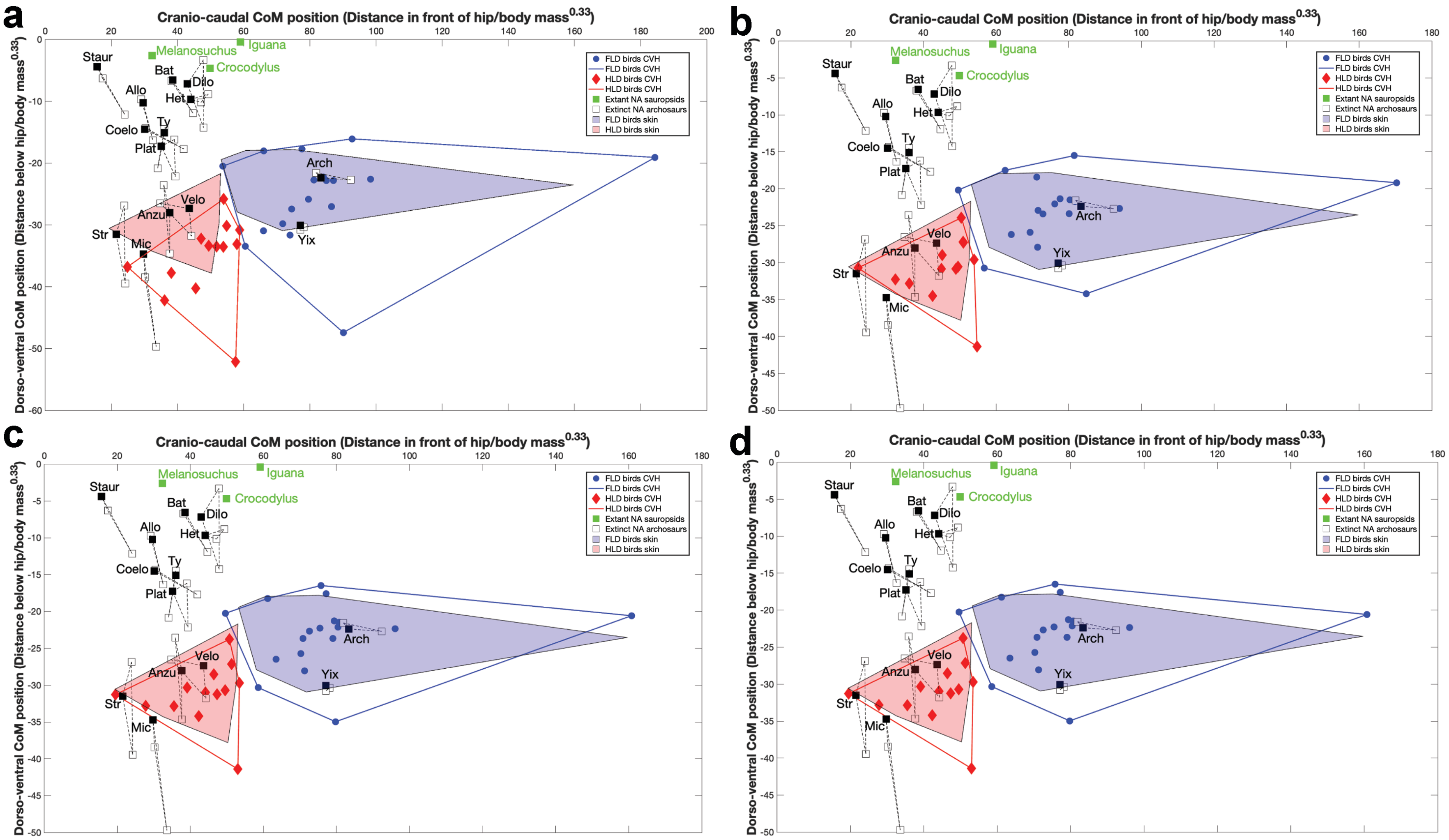

Supplementary Figure 9. Comparison of CoM positions measured from skin volume models in HLD (red shaded zone) and FLD (blue shaded zones) to those predicted when birds are treated as fossil animals and their CoMs predicted by different expansions of convex hull volumes, specifically **a.** the average body segment expansion factors given extant non-avian saurians and birds, **b.** the average body segment expansion factors given by birds-only, **c.** the allometric expansion equations for individual body segments given by extant non-avian saurians and birds, and **d.** the allometric expansion equations for individual body segments given by extant and birds-only. Source data are provided as a Source Data file.

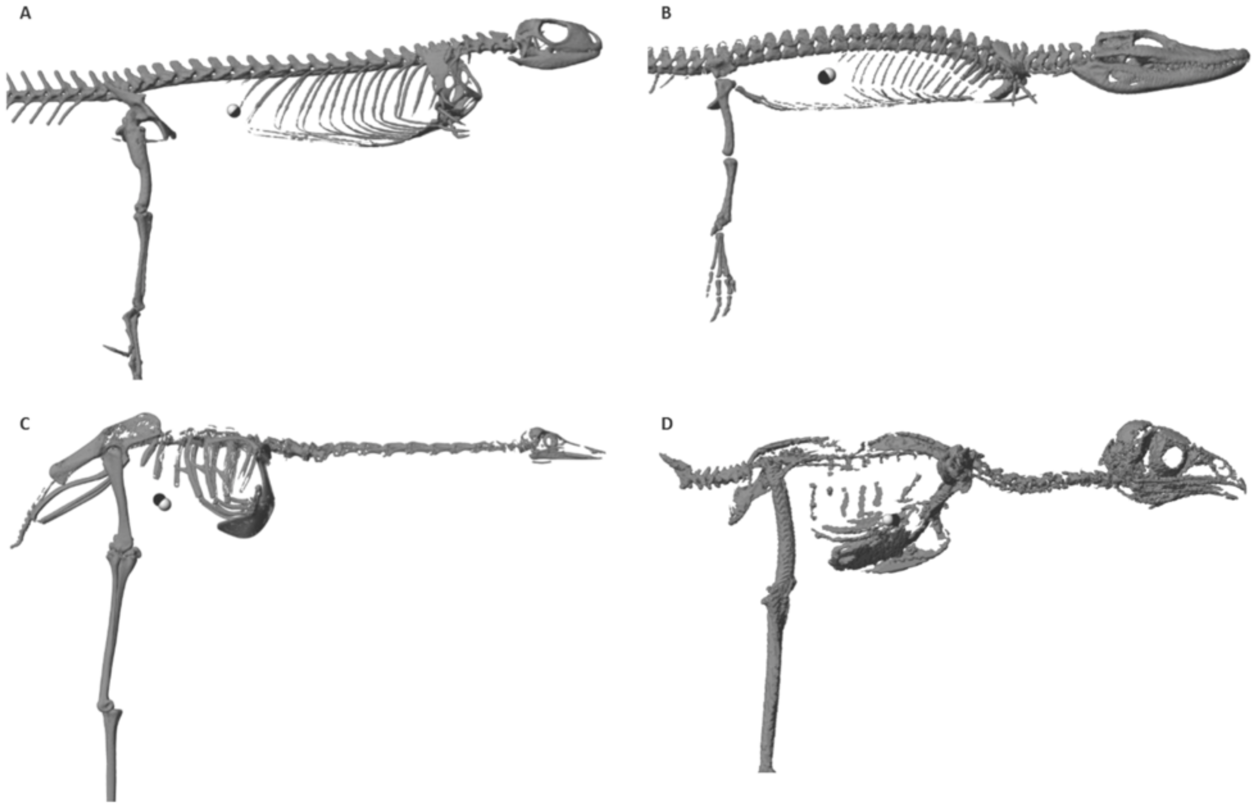

Supplementary Figure 10. Differences between whole body CoMs derived from skeletal segment CoMs (white spheres) and skin segment CoMs (black spheres), shown in the context of each specimen (**A**: iguana, **B**: alligator, **C**: rhea, **D**: buzzard).

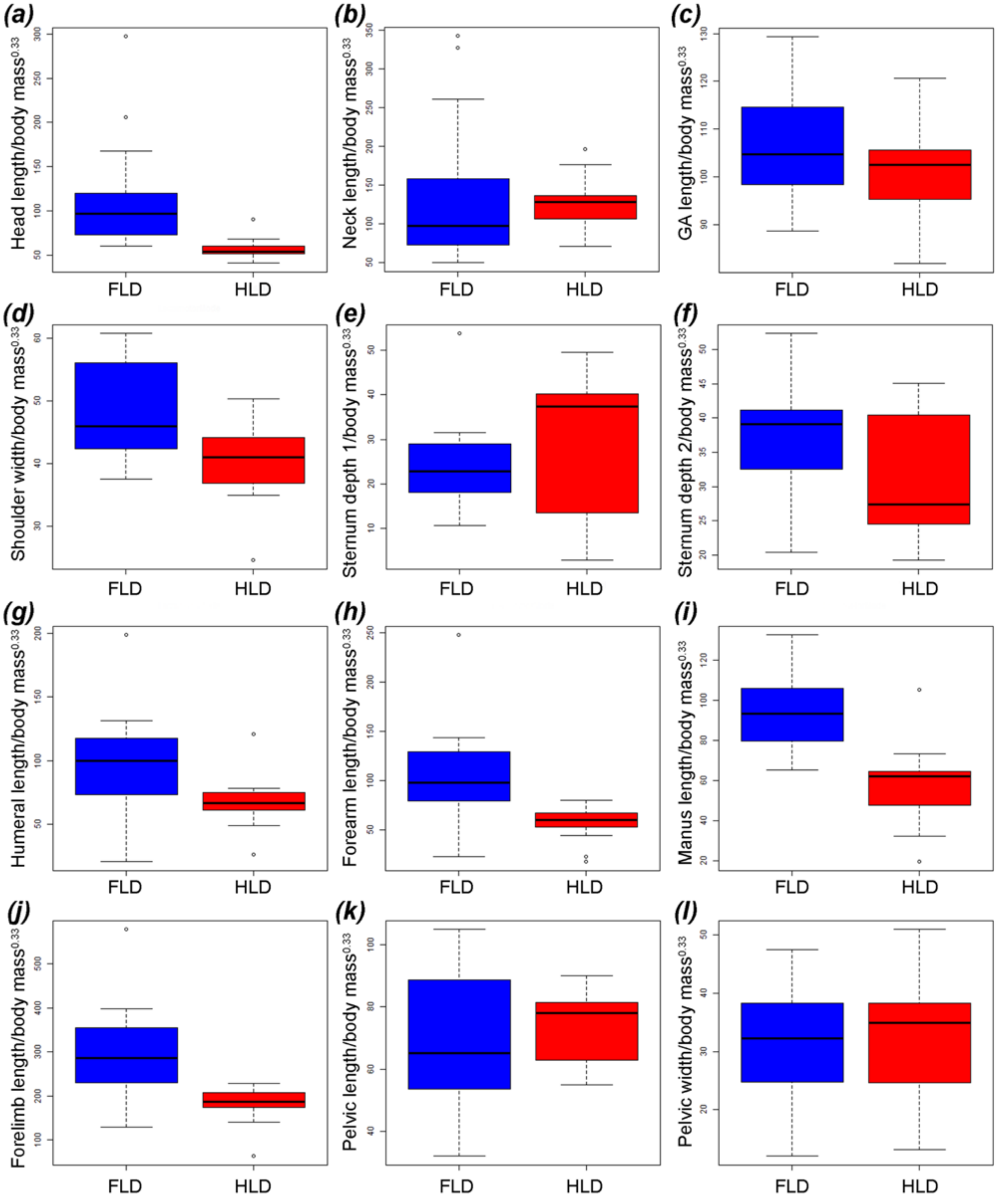

Figure continues overleaf…

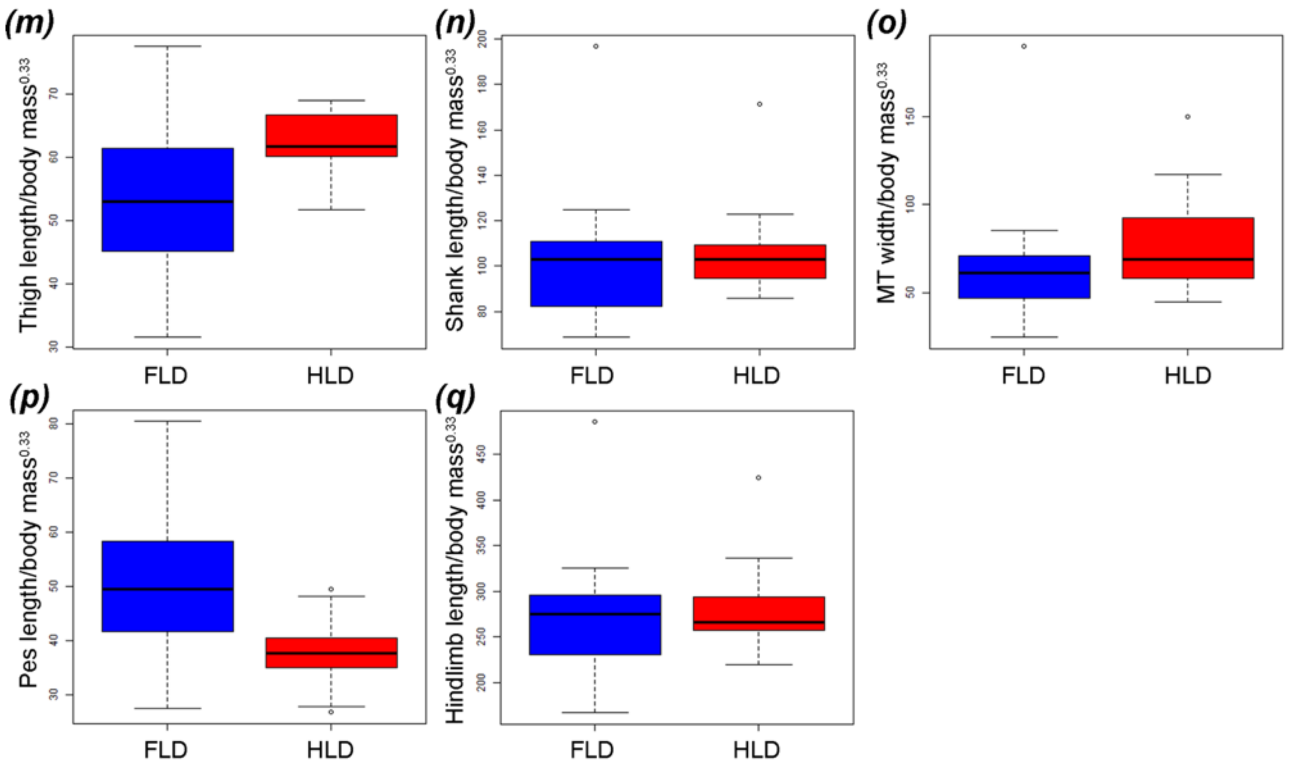

Supplementary Figure 11. Comparison of size-normalised segment lengths and other linear dimensions in forelimb-dominated (FLD, blue) and hindlimb-dominated (HLD, red) birds. Dimensions compared are ***(a)*** head length, ***(b)*** neck length, ***(c)*** gleno-acetabular (GA) length, ***(d)*** shoulder width, ***(e)*** sternum depth 1 and ***(f)*** 2 (see landmarks on Supplementary Figure 1), ***(g)*** humeral length, ***(h)*** forearm length, ***(i)*** manus length, ***(j)*** forelimb length, ***(k)*** pelvic length, ***(l)*** pelvic width, ***(m)*** thigh length, ***(n)*** shank length, ***(o)*** metatarsal (MT) length, ***(p)*** pes length and ***(q)*** hindlimb length. Statistical comparisons using two-sided pANOVAs indicate that FLD birds have significantly (P = <0.05) greater skull lengths (P = 0.002), shoulder widths (P = 0.008), sternum depth 2 (P = 0.038), humeral lengths (P = 0.012), forearm lengths (P = 0.002), manus lengths (P = 0.002), forelimb lengths (P = 0.002) and pes lengths (P = 0.004), and significantly lower thigh lengths (P = 0.018) for their size than HLD birds. Differences between other parameters were not statistically significant (P = >0.05). In each boxplot, the horizontal black line indicates the median values and the bottom and top edges of the box indicate the 25th and 75th percentiles, respectively. The whiskers extend to the most extreme data points not considered outliers, and the outliers are plotted individually as hollow circles. For FLD birds n=20 independent species and for HLD birds n=13 independent species. Source data are provided as a Source Data file.

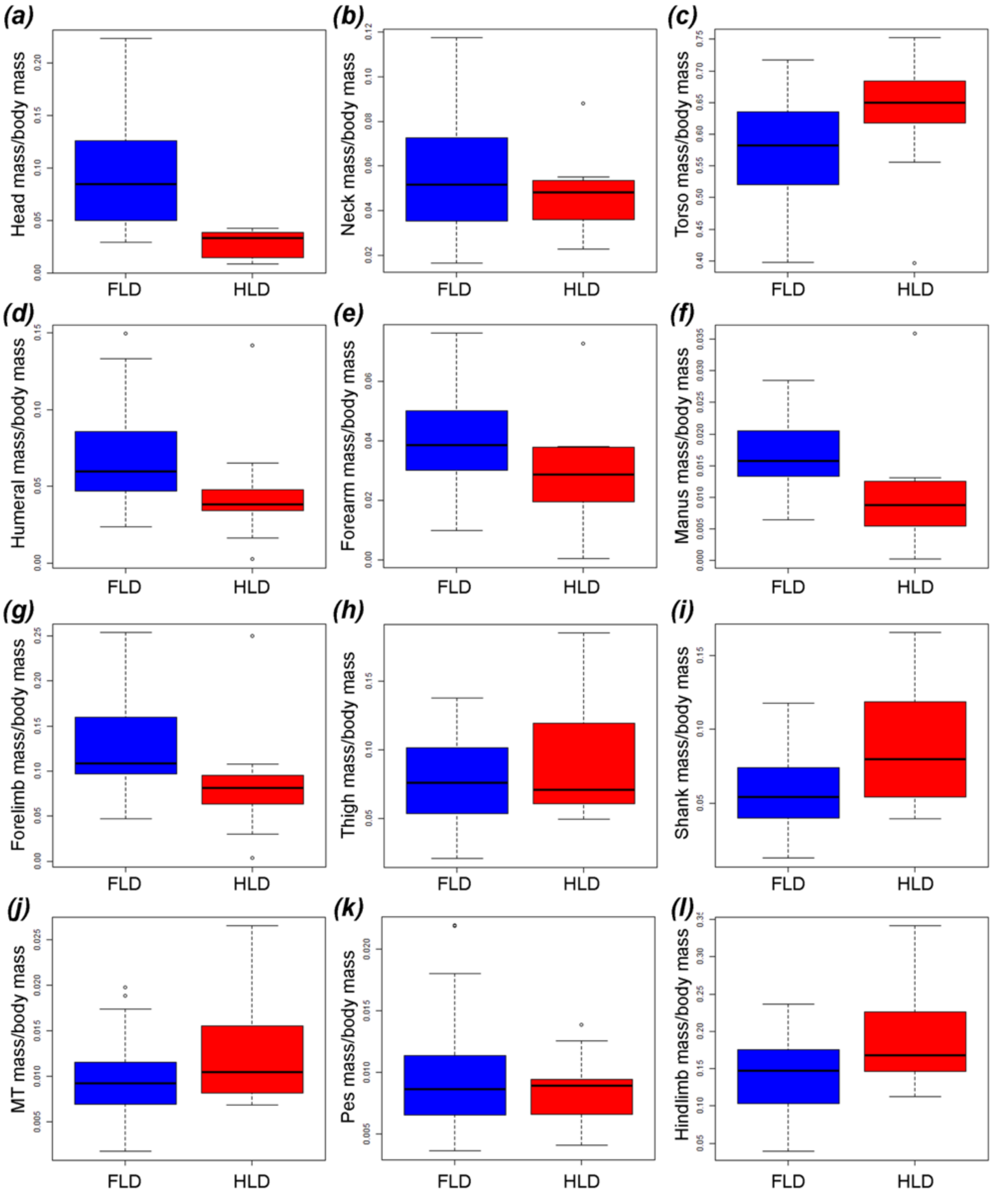

Supplementary Figure 12. Comparison of size-normalised segment masses forelimb-dominated (FLD, blue) and hindlimb-dominated (HLD, red) birds. Dimensions compared are relative ***(a)*** head. ***(b)*** neck, ***(c)*** torso, ***(d)*** humeral, ***(e)*** forearm, ***(f)*** manus, ***(g)*** forelimb, ***(h)*** thigh, ***(i)*** shank, ***(j)*** metatarsal (MT), ***(k)*** pes and ***(l)*** hindlimb segment masses. Statistical comparisons using two-sided pANOVAs indicate that FLD birds have significantly (P = <0.05) greater head (P = 0.002), humeral (P = 0.049), manus (P = 0.024) and forelimb (P = 0.03) segment masses, and significantly lower shank (P = 0.024) and hindlimb (P = 0.024) masses for their size than HLD birds. Differences between other parameters were not statistically significant (P = >0.05). For FLD birds n=20 independent species and for HLD birds n=13 independent species. In each boxplot, the horizontal

black line indicates the median values and the bottom and top edges of the box indicate the 25th and 75th percentiles, respectively. The whiskers extend to the most extreme data points not considered outliers, and the outliers are plotted individually as hollow circles. Source data are provided as a Source Data file.

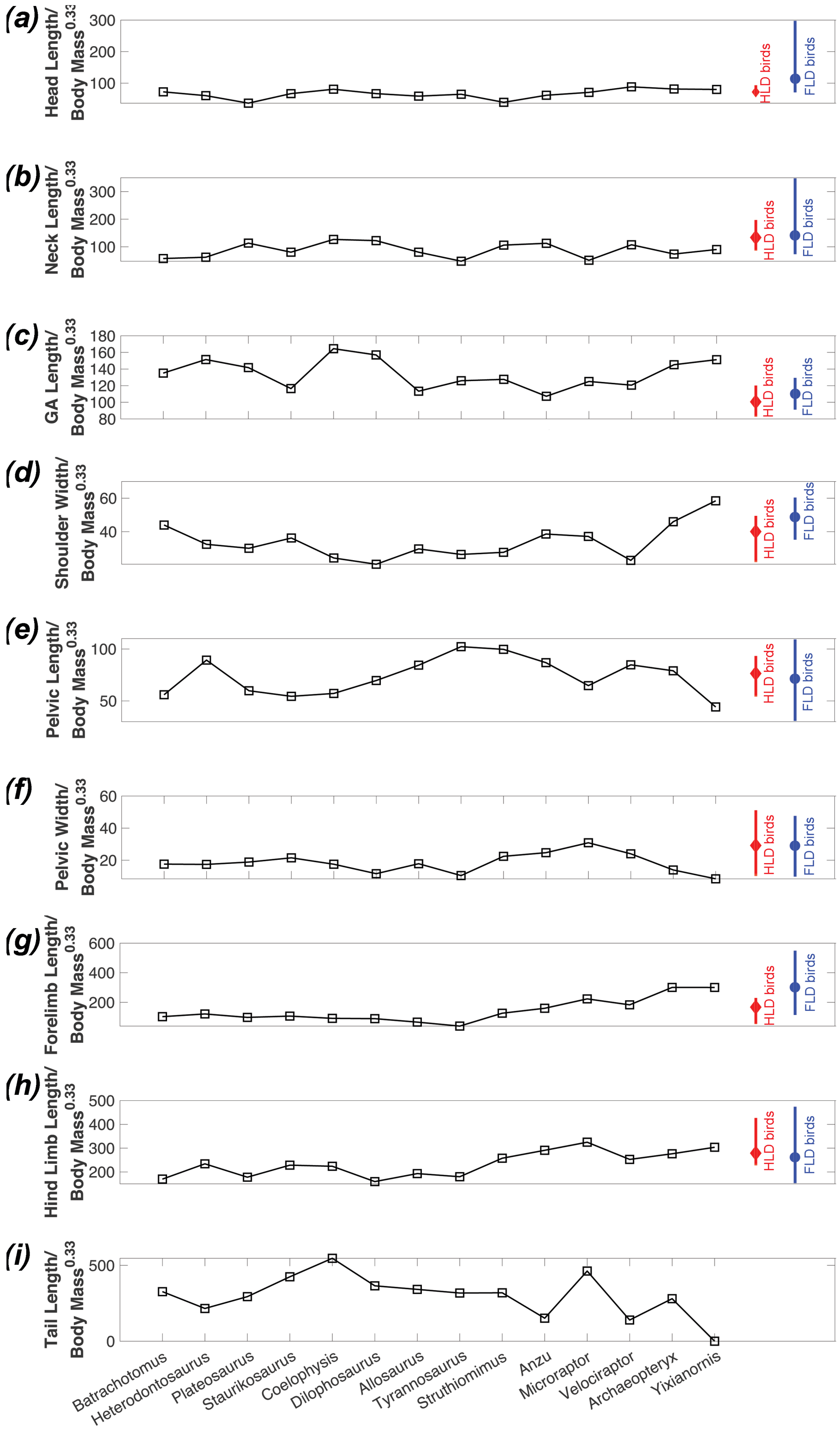

Supplementary Figure 13. Normalised linear body segment proportions in archosaurian fossils, with comparison to the mean values (red diamond, blue circle) and ranges (red and blue bars) measured in n=13 extant hind limb dominated (HLD) and n=20 forelimb dominated (FLD) birds. Parameters compared are normalised (a) head length, (b) neck length, (c) gleno-acetabular (GA) distance, (d) shoulder width, (e) pelvic length, (f) pelvic length, (g) forelimb length, (h) hindlimb length and (i) tail length. The most striking trends are observed in (d) shoulder width, (g) forelimb

length and (i) tail length with the early avialan birds *Archaeopteryx* and *Yixianornis* attaining relative segment lengths consistent with extant FLD birds. Source data are provided as a Source Data file.

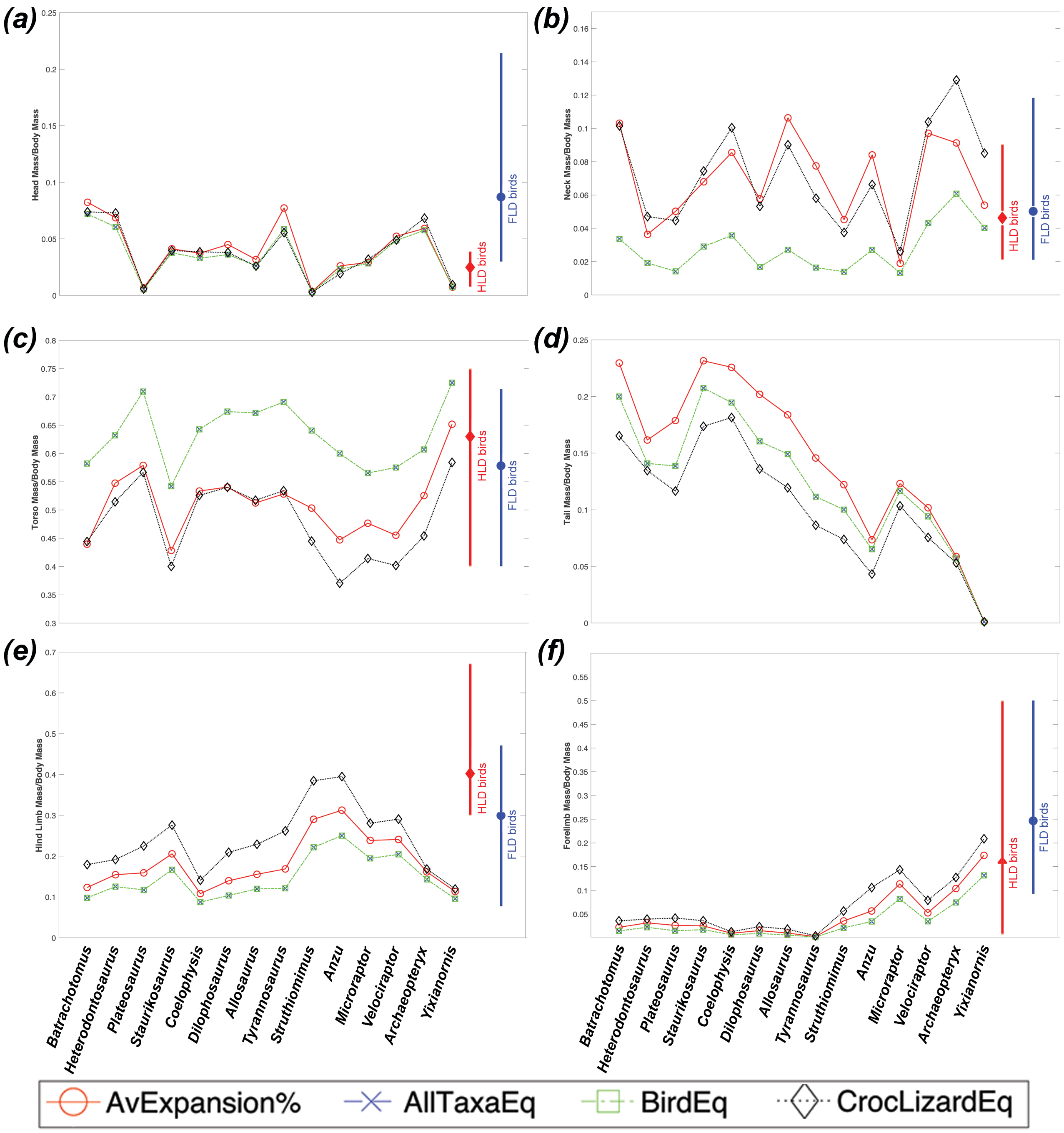

Supplementary Figure 14. Predicted normalised body segment mass evolution in archosaurian fossils, with comparison to mean values (red diamond, blue circle) and ranges (red and blue bars) measured in n=13 extant hind limb dominated (HLD) and n=20 forelimb dominated (FLD) birds. Parameters compared are normalised (a) head, (b) neck, (c) torso, (d) tail, (e) hindlimb and (f) torso mass. The most striking trends are observed in the (e-f) limb (d) and tail segments, with (e) hind limb and (d) tail mass decreasing and (f) forelimb mass increasing in crownward Maniraptoriformes, ultimately resulting in early avialan birds like *Archaeopteryx* and *Yixianornis* attaining segment proportions and overall centre of mass positions (Fig. 2) consistent with extant FLD birds. Source data are provided as a Source Data file.

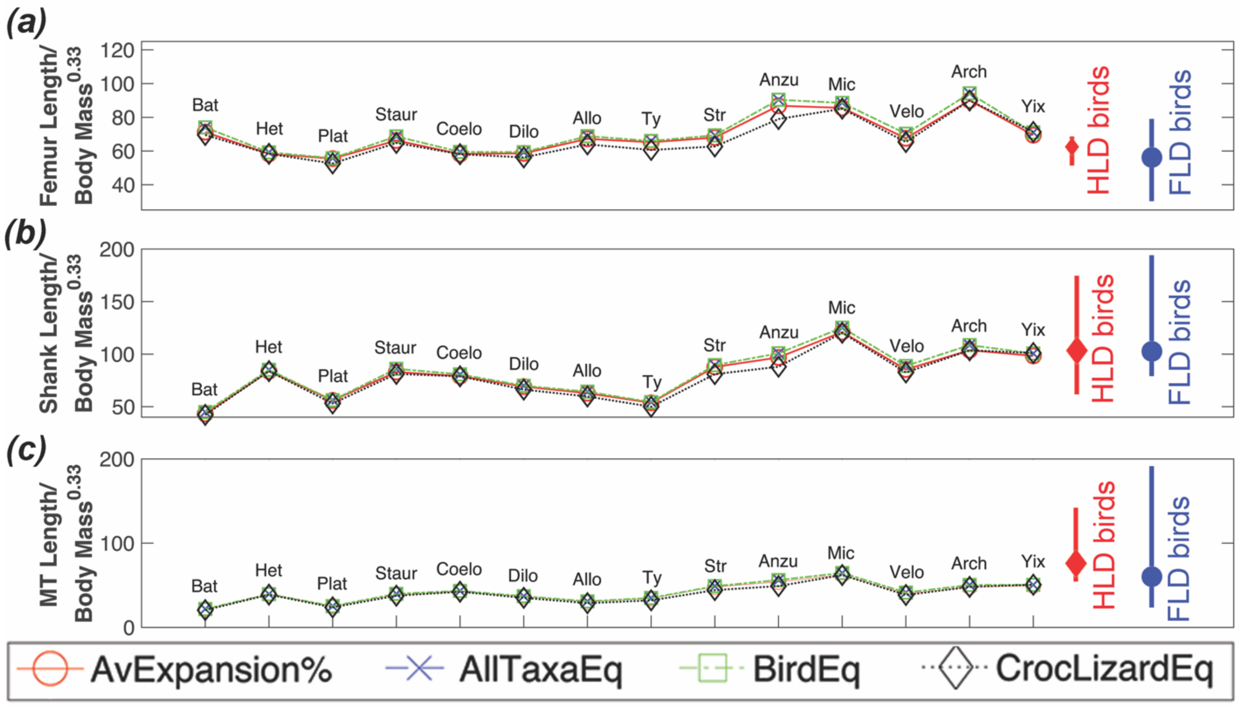

Supplementary Figure 15. Changes in the relative lengths of the (a) femur, (b) shank and (c) metatarsal (MT) segments in archosaurian taxa, with comparison to the mean values (red diamond, blue circle) and ranges (red and blue bars) measured in n=13 extant hindlimb dominated (HLD) and n=20 forelimb dominated (FLD) birds. Source data are provided as a Source Data file.

Supplementary Tables

Supplementary Table 1. Differences between skeletal hull CoM and skin CoM for four specimens. All measurements in mm

A		Head	Neck	Torso	Tail	Arm	Forearm	Hand	Thigh	Shank	MT	Toes
**Iguana**	**x**	0.0	2.0	0.0	-0.1	-5.0	-1.0	2.2	0.0	-1.0	0.7	1.0
	**y**	1.0	6.0	0.0	2.8	0.0	-1.0	-0.2	-4.0	5.0	-0.6	-0.7
	**z**	-1.0	1.0	2.0	-1.9	-1.0	1.0	0.5	-3.0	-2.0	-0.4	-0.5
**Alligator**	**x**	0.4	0.0	-1.0	-0.1	-2.0	2.0	-0.2	-1.0	-2.0	-0.7	-1.1
	**y**	1.5	6.0	0.0	1.6	-1.0	0.0	0.5	1.0	1.0	1.9	-0.2
	**z**	-0.5	0.0	0.0	-6.6	-1.0	1.0	1.1	-2.0	-1.0	-0.7	-1.4
**Rhea**	**x**	-0.8	-0.2	0.0	-1.0	26.0	9.0	5.0	7.0	-2.0	0.0	0.1
	**y**	0.3	0.3	7.0	2.0	-1.0	1.0	2.0	-35.0	-25.0	3.0	0.6
	**z**	-2.4	48.9	-17.0	12.0	-5.0	-2.0	-1.0	0.0	3.0	-3.0	-0.6
**Buzzard**	**x**	0.0	-2.4	-2.0	-3.0	20.0	13.0	3.0	1.0	-1.0	-1.0	0.0
	**y**	-1.0	2.3	2.0	2.0	1.0	0.0	1.0	-6.0	-6.0	-6.0	0.4
	**z**	-2.0	0.4	7.0	2.0	3.0	-1.0	0.0	-4.0	-1.0	0.0	0.0

Supplementary Table 2. Whole body CoMs derived from skeletal segment CoMs and skin segment CoMs, with 3D distances between the two estimates. Where +x is right, +y is dorsal and +z is caudal. All measurements in mm.

		Skeletal CoM	Skin CoM	3D distance
**Iguana**	**x**	-0.5	-0.5	0.849
	**y**	0.6	0.0	
	**z**	-60.4	-61	
**Alligator**	**x**	-12.7	-12.2	2.035
	**y**	0.6	-0.4	
	**z**	-50.2	-48.5	
**Rhea**	**x**	6.4	4.4	10.808
	**y**	-78.7	-71.6	
	**z**	-77.7	-69.8	
**Buzzard**	**x**	5.3	4.4	3.134
	**y**	-26.3	-26.2	
	**z**	-59.8	-62.8	

Supplementary Table 3. Occurrence ranges (first and last appearances) for fossil taxa used in our phylogenetic analysis. Where possible we have used geochronological estimates of parent formations for our FADLAD (n = 6), however this was not possible for 7 taxa, which have instead been time-scaled using the upper and lower boundaries of the geologic substages in which they occur.

**Taxon**	**FAD**	**LAD**	**Reference**	**Notes**
Allosaurus jimmadseni	157.32	152.77	Chure & Loewen[15]	Based on geochronology (see Chure & Loewen[15]).
Anzu wyliei	68.196	66.04	Lamanna *et al.*[16]	Based on geochronology (Fowler[17])
Archaeopteryx lithographica	149.24	143.1	Schweigert[18]	Tithonian
Batrachotomus kupferzellensis	239.48	237	Gower & Schoch[19]	Ladinian
Coelophysis bauri	213.124	207.8	Speilmann *et al.*[20]	Based on geochronology (Ramezani *et al.*[21])
Dilophosaurus wetherilli	201.36	192.9	Marsh & Rowe[5]	Hettangian & Sinemurian
Heterodontosaurus tucki	199.46	184.2	Norman *et al.*[22]	Sinemurian & Pleinsbachian
Microraptor gui	122	118.9	Xu *et al.*[23]	Based on geochronology (Yu *et al.*[24])
Plateosaurus trossingensis	227.3	201.36	Lallensack *et al.*[25]	Norian & Rhaetian, previously *P. engelhardti* (see Lallensack *et al.*[25]).
Staurikosaurus pricei	237	227.3	Bittencourt & Kellner[26]	Carnian
Struthiomimus sedens	83.65	66.04	Longrich[27]	Campanian & Maastrichtian
Tyrannosaurus rex	68.196	66.04	Larson & Carpenter[28]	Based on geochronology (Fowler[17])
Velociraptor mongoliensis	83.65	72.17	Godefroit *et al.*[29]	Campanian
Yixianornis grabaui	122	118.9	Zhou & Zhang[30]	Based on geochronology (Yu *et al.*[24])

Supplementary Table 4. Body segment and whole-body masses and centre-of-mass values for the *Anzu* unexpanded convex hull model at the scale used by Allen et al.[14]. The size-normalised CoM prediction is nearly identical to the model re-scaled isometrically here (Supplementary Table 39), as should occur given uniform expansion of the whole skeletal model.

	Volume	Density	Mass	CMX	CMY
Head	0.000298	1000	0.298	0.5677	0.0710
Neck	0.000219	1000	0.219	0.3306	0.0517
Thoracic	0.005136	1000	5.136	0.0999	-0.0081
Tail	0.000283	1000	0.283	-0.1860	-0.0269
Humerus	0.000029	1000	0.029	0.2470	0.0072
Forearm	0.000022	1000	0.022	0.2475	0.0066
Hand	0.000101	1000	0.101	0.2410	0.0116
Humerus	0.000029	1000	0.029	0.2470	0.0072
Forearm	0.000022	1000	0.022	0.2475	0.0066
Hand	0.000101	1000	0.101	0.2410	0.0116
Thigh	0.000238	1000	0.238	0.0010	-0.0791
Shank	0.000235	1000	0.235	0.0017	-0.2944
MTs	0.000104	1000	0.104	-0.0003	-0.4832
Foot	0.000065	1000	0.065	-0.0034	-0.5806
Thigh	0.000238	1000	0.238	0.0010	-0.0791
Shank	0.000235	1000	0.235	0.0017	-0.2944
MTs	0.000104	1000	0.104	-0.0003	-0.4832
Foot	0.000065	1000	0.065	-0.0034	-0.5806
Trunk Mass	0.005936	1000	5.936	0.1183	-0.0028
Hindlimb Mass	0.000642	1000	0.642	0.0006	-0.2741
Forelimb Mass	0.000152	1000	0.152	0.2431	0.0100
Total Body Mass	0.007524	1000	7.524	0.1032	-0.0486
Normalised CoM prediction				0.053	-0.025

Supplementary Table 5. Body segment and whole-body masses and centre-of-mass values for the *Anzu* unexpanded convex hull model after re-scaling of the skeletal model. The size normalised CoM prediction is nearly identical to the model at the scale used by Allen et al.[14] (Supplementary Table 38), as should occur given uniform expansion of the whole skeletal model.

	**Volume**	**Density**	**Mass**	**CMX**	**CMY**
Head	0.008042	1000	8.042	1.703	0.213
Neck	0.005897	1000	5.897	0.991	0.155
Thoracic	0.138669	1000	138.669	0.300	-0.024
Tail	0.007631	1000	7.631	-0.558	-0.081
Humerus	0.000791	1000	0.791	0.741	0.022
Forearm	0.000594	1000	0.594	0.742	0.020
Hand	0.002715	1000	2.715	0.723	0.149
Humerus	0.000791	1000	0.791	0.741	0.022
Forearm	0.000594	1000	0.594	0.742	0.020
Hand	0.002715	1000	2.715	0.723	0.149
Thigh	0.006436	1000	6.436	0.003	-0.237
Shank	0.00634	1000	6.34	0.005	-0.883
MTs	0.002799	1000	2.799	-0.001	-1.449
Foot	0.001765	1000	1.765	-0.010	-1.743
Thigh	0.006436	1000	6.436	0.003	-0.237
Shank	0.00634	1000	6.34	0.005	-0.883
MTs	0.002799	1000	2.799	-0.001	-1.449
Foot	0.001765	1000	1.765	-0.010	-1.743
Trunk Mass	0.160239	1000	160.239	0.355	-0.008
Hindlimb Mass	0.01734	1000	17.34	0.002	-0.822
Forelimb Mass	0.0041	1000	4.1	0.729	0.106
Total Body Mass	0.203119	1000	203.119	0.310	-0.143
Normalised CoM prediction				0.054	-0.025

Supplementary Table 6. Body segment and whole-body masses and centre-of-mass values for the *Archaeopteryx* unexpanded convex hull model at the scale used by Allen et al.[14]. The size normalised CoM prediction is nearly identical to the model re-scaled isometrically here (Supplementary Table 41), as should occur given uniform expansion of the whole skeletal model.

	Volume	Density	Mass	CMX	CMY
Head	4.63834E-06	1000	0.0046	0.1214	0.0011
Neck	1.62976E-06	1000	0.0016	0.0882	-0.0005
Thoracic	2.65337E-05	1000	0.0265	0.0353	-0.0066
Tail	1.55247E-06	1000	0.0016	-0.0528	-0.0007
Humerus	6.36886E-07	1000	0.0006	0.0645	-0.0005
Forearm	5.42213E-07	1000	0.0005	0.0650	-0.0410
Hand	7.56594E-07	1000	0.0008	0.0639	-0.0007
Humerus	6.36886E-07	1000	0.0006	0.0645	-0.0005
Forearm	5.42213E-07	1000	0.0005	0.0650	-0.0410
Hand	7.56594E-07	1000	0.0008	0.0639	-0.0007
Thigh	9.35922E-07	1000	0.0009	0.0003	-0.0194
Shank	8.32556E-07	1000	0.0008	0.0000	-0.0580
MTs	1.77501E-07	1000	0.0002	0.0001	-0.0924
Foot	2.22103E-07	1000	0.0002	-0.0005	-0.1097
Thigh	9.35922E-07	1000	0.0009	0.0003	-0.0194
Shank	8.32556E-07	1000	0.0008	0.0000	-0.0580
MTs	1.77501E-07	1000	0.0002	0.0001	-0.0924
Foot	2.22103E-07	1000	0.0002	-0.0005	-0.1097
Trunk Mass	3.43543E-05	1000	0.0344	0.0454	-0.0050
Hindlimb Mass	2.16808E-06	1000	0.0022	0.0001	-0.0494
Forelimb Mass	1.93569E-06	1000	0.0019	0.0644	-0.0119
Total Body Mass	4.25618E-05	1000	0.0426	0.0425	-0.0102
Normalised CoM prediction				0.121	-0.0288

Supplementary Table 7. Body segment and whole-body masses and centre-of-mass values for the *Archaeopteryx* unexpanded convex hull model after re-scaling of the skeletal model. The size normalised CoM prediction is nearly identical to the model at the scale used by Allen et al.[14] (Supplementary Table 40), as should occur given uniform expansion of the whole skeletal model.

	Volume	Density	Mass	CMX	CMY	CMZ
Head	1.56541E-05	1000	0.0157	0.182	0.002	0
Neck	5.50056E-06	1000	0.0055	0.132	-0.001	0
Thoracic	0.000138	1000	0.138	0.053	-0.013	0
Tail	5.2394E-06	1000	0.0052	-0.079	-0.001	0
Humerus	2.14948E-06	1000	0.0021	0.097	-0.001	-0.05623
Forearm	1.82995E-06	1000	0.0018	0.097	-0.001	-0.11708
Hand	2.5535E-06	1000	0.0026	0.096	-0.001	-0.18036
Humerus	2.14948E-06	1000	0.0021	0.097	-0.001	-0.05623
Forearm	1.82995E-06	1000	0.0018	0.097	-0.001	-0.11708
Hand	2.5535E-06	1000	0.0026	0.096	-0.001	-0.18036
Thigh	3.15874E-06	1000	0.0032	0.000	-0.029	-0.01729
Shank	2.80995E-06	1000	0.0028	0.000	-0.088	-0.01939
MTs	5.99042E-07	1000	0.0006	0.000	-0.139	-0.01683
Foot	7.49625E-07	1000	0.0007	-0.001	-0.165	-0.01498
Thigh	3.15874E-06	1000	0.0032	0.000	-0.029	-0.01729
Shank	2.80995E-06	1000	0.0028	0.000	-0.088	-0.01939
MTs	5.99042E-07	1000	0.0006	0.000	-0.139	-0.01683
Foot	7.49625E-07	1000	0.0007	-0.001	-0.165	-0.01498
Trunk Mass	0.000164	1000	0.164	0.068	-0.008	0
Hindlimb Mass	7.31736E-06	1000	0.0073	0.000	-0.075	
Forelimb Mass	6.53293E-06	1000	0.0065	0.097	-0.001	
Total Body Mass	0.000192	1000	0.192	0.064	-0.014	0
Normalised CoM prediction				0.111	-0.026	

Supplementary References

1. Jetz, W., Thomas, G. H., Joy, J. B., Hartmann, K. and Mooers, A. O. 2012. The global diversity of birds in space and time. *Nature*, **491**, 444-448.
2. Ezcurra, M. D. and Brusatte, S. L. 2011. Taxonomic and phylogenetic reassessment of the early neotheropod dinosaur Camposaurus arizonensis from the Late Triassic of North America. *Palaeontology*, **54**, 763-772.
3. Carrano, M. T., Benson, R. B. and Sampson, S. D. 2012. The phylogeny of tetanurae (Dinosauria: Theropoda). *Journal of Systematic Palaeontology*, **10**, 211-300.
4. Brusatte, S. L., Lloyd, G. T., Wang, S. C. and Norell, M. A. 2014. Gradual assembly of avian body plan culminated in rapid rates of evolution across the dinosaur-bird transition. *Current Biology*, **24**, 2386-2392.
5. Marsh, A. D. and Rowe, T. B. 2020. A comprehensive anatomical and phylogenetic evaluation of *Dilophosaurus wetherilli* (Dinosauria, Theropoda) with descriptions of new specimens from the Kayenta Formation of northern Arizona. *Journal of Paleontology*, **94**, 1-103.
6. Pei, R., Pittman, M., Goloboff, P. A., Dececchi, T. A., Habib, M. B., Kaye, T. G., Larsson, H. C., Norell, M. A. Brusatte, S. L. and Xu, X. 2020. Potential for powered flight neared by most close avialan relatives, but few crossed its thresholds. *Current Biology*, **30**, 4033-4046.
7. Bapst, D. W. 2012. paleotree: an R package for paleontological and phylogenetic analyses of evolution. *Methods in Ecology and Evolution*, **3**, 803-807.
8. Bapst, D. W. 2013. A stochastic rate-calibrated method for time-scaling phylogenies of fossil taxa. *Methods in Ecology and Evolution*, **4**, 724-733.
9. Gradstein, F. M., Ogg, J. G., Schmitz, M. D. and Ogg, G. M. 2020. *Geologic time scale 2020*. Elsevier, Amsterdam, Netherlands, 1300pp.
10. Darlim, G., Lee, M. S., Walter, J. and Rabi, M. 2022. The impact of molecular data on the phylogenetic position of the putative oldest crown crocodilian and the age of the clade. *Biology Letters*, **18**, 20210603.

11. Gemmel, N. J., Rutherford, K., Prost, S., Tollis, M., Winter, D., Macey, J. R., Adelson, D. L., Suh, A., Bertozzi, T., Grau, J. H. and Organ, C. 2020. The tuatara genome reveals ancient features of amniote evolution. *Nature*, **584**, 403-409.
12. Castiglione, S., Tesone, G., Piccolo, M., Melchionna, M., Mondanaro, A., Serio, C., Febbraro, M. D. and Raia, P. 2018. A new method for testing evolutionary rate variation and shifts in phenotypic evolution. *Methods in Ecology and Evolution*, **9**, 974-983.
13. Revell, L. J. 2012. phytools: an R package for phylogenetic comparative biology (and other things). *Methods in ecology and evolution*, **2**, 217-223.
14. Allen, V., Bates, K.T., Li, Z. & Hutchinson, J.R. Linking the evolution of body shape and locomotor biomechanics in bird-line archosaurs. Nature 497, 104-108 (2013).
15. Chure, D. J. and Loewen, M. A. 2020. Cranial anatomy of Allosaurus jimmadseni, a new species from the lower part of the Morrison Formation (Upper Jurassic) of Western North America. *PeerJ*, **8**, e7803.
16. Lamanna, M. C., Sues, H. D., Schachner, E. R. and Lyson, T. R. 2014. A new large-bodied oviraptorosaurian theropod dinosaur from the latest Cretaceous of western North America. *PloS one*, **9**, e92022.
17. Fowler, D. 2020. The Hell Creek Formation, Montana: A stratigraphic review and revision based on a sequence stratigraphic approach. *Geosciences,* **10**, 435.
18. Schweigert, G. 2007. Ammonite biostratigraphy as a tool for dating Upper Jurassic lithographic limestones from South Germany – first results and open questions. *Neues Jahrbuch für Geologie und Paläontologie-Abhandlungen*, **245**, 117-125.
19. Gower, D. J. and Schoch, R. R. 2009. Postcranial anatomy of the rauisuchian archosaur *Batrachotomus kupferzellensis*. *Journal of Vertebrate Paleontology*, **29**, 103-122.
20. Spielmann, J. A., Lucas, S. G., Rinehart, L. F., Hunt, A. P., Heckert, A. B. and Sullivan, R. M. 2007. Oldest records of the Late Triassic theropod dinosaur *Coelophysis bauri. New Mexico Museum of Natural History and Science Bulletin*, **41**, 384-401.
21. Ramezani, J., Fastovsky, D. E. and Bowring, S. A. 2014. Revised chronostratigraphy of the lower Chinle Formation strata in Arizona and New Mexico (USA): high-precision U-Pb geochronological constraints on the Late Triassic evolution of dinosaurs. *American Journal of Science*, **314**, 981-1008.
22. Norman, D. B., Crompton, A. W., Butler, R. J., Porro, L. B. and Charig, A. J. 2011. The Lower Jurassic ornithischian dinosaur *Heterodontosaurus tucki* Crompton & Charig, 1962: cranial anatomy, functional morphology, taxonomy, and relationships. *Zoological Journal of the Linnean Society*, **162**, 182-276.

23. Xu, X., Zhou, Z., Wang, X., Kuang, X., Zhang, F. and Du, X. 2003. Four-winged dinosaurs from China. *Nature*, **421**, 335-340.
24. Yu, Z., Wang, M., Li, Y., Deng, C. and He, H. 2021. New geochronological constraints for the Lower Cretaceous Jiufotang Formation in Jianchang Basin, NE China, and their implications for the late Jehol Biota. *Palaeogeography, Palaeoclimatology, Palaeoecology*, **583**, 110657.
25. Lallensack, J. N., Teschner, E., Pabst, B. and Sander, M. P. 2021. New skulls of the basal sauropodmorph *Plateosaurus trossingensis* from Frick, Switzerland: Is there more than one species? *Acta Palaeontologica Polonica*, 66, 1-28.
26. Bittencourt, J. D. S. and Kellner, A. W. A. 2009. The anatomy and phylogenetic position of the Triassic dinosaur *Staurikosaurus pricei* Colbert, 1970. *Zootaxa*, **2079**, 1-56.
27. Longrich, N. 2008. A new, large ornithomimid from the Cretaceous Dinosaur Park Formation of Alberta, Canada: implications for the study of dissociated dinosaur remains. *Palaeontology*, **51**, 983-997.
28. Larson, P. L. and Carpenter, K. 2008. *Tyrannosaurus rex*, the tyrant king. Indian University Press, Bloomington, IN, 438pp.
29. Godefroit, P., Currie, P. J., Hong, L., Yong, S. C. and Zhi-Ming, D. 2008. A new species of *Velociraptor* (Dinosauria: Dromaeosauridae) from the Upper Cretaceous of northern China. *Journal of Vertebrate Paleontology*, **28**, 432-438.
30. Zhou, Z. and Zhang, F. 2001. Two new ornithurine birds from the Early Cretaceous of western Liaoning, China. *Chinese Science Bulletin*, **46**, 1258-1264.
