# Supplementary material for: Decoupling body shape and mass distribution in birds and their dinosaurian ancestors
Source: Nat Commun. 2023 Mar 22;14:1575. doi: 10.1038/s41467-023-37317-y (PMC10033513; doi:10.1038/s41467-023-37317-y)
Supplement: Supplementary file 2 — Description of Additional Supplementary Files [file 41467_2023_37317_MOESM2_ESM.pdf]

Supplementary Data 1: List of extant birds used in current study, including classification as forelimb (FLD) or hind limb (HLD) dominated locomotion and body mass (kg).

Supplementary Data 2: Extant non-avian sauropsids included in the current study.

Supplementary Data 3: All taxa predictive equations (convex hull volume vs. skin volume).

Supplementary Data 4: Bird-only predictive equations (convex hull volume vs. skin volume).

Supplementary Data 5: Non-avian sauropsid predictive equations (convex hull volume vs. skin volume).

Supplementary Data 6: Average expansion factors (convex hull:skin volume).

Supplementary Data 7: Statistical comparison of crano-caudal (CC) and dorso-ventral (DV) CoM position in HLD vs FLD birds, in models where CoMs are calculated with heterogenous segment densities.

Supplementary Data 8: Body mass variation between FLD and HLD birds (full data set and with ratites and the pelican removed): generalised least squares fit by REML.

Supplementary Data 9: Summary of linear models of CoM vs body mass for all birds, in models with heterogeneous segment densities.

Supplementary Data 10: Summary of linear models of CoM vs body mass for HLD birds, in models with heterogeneous segment densities.

Supplementary Data 11: Summary of linear models fo CoM vs body mass for FLD birds (full data set and with pelican removed), in models with heterogeneous segment densities.

Supplementary Data 12: Summary of linear models CoM vs body mass for HLD birds with ratites removed, in models with heterogeneous segment densities.

Supplementary Data 13: PhylANCOVA comparisons for CC and DV CoM positions in HLD vs FLD birds.

Supplementary Data 14: CC_CoM vs linear segment parameters, with CoM calculated with heterogeneous segment density.

Supplementary Data 15: DV_CoM vs linear segment parameters, with CoM calculated with heterogeneous segment density.

Supplementary Data 16: CC_CoM vs segment masses, with CoM calculated with heterogeneous segment density.

Supplementary Data 17: DV_CoM vs mass segment parameters, with CoM calculated with heterogeneous segment density.

Supplementary Data 18: Differences in linear segment properties between HLD and FLD birds tested with two-sided pANOVA, including an iteration with the pelican removed.

Supplementary Data 19: Differences in mass segment properties between HLD and FLD birds tested with two-sided pANOVA, including an iteration with the pelican removed.

Supplementary Data 20: Results of Spearman's rank correlations between normalised body segment linear properties and masses and CC_CoM position in bird-line taxa using raw taxon values.

Supplementary Data 21: Results of Spearman's rank correlations between normalised body segment linear properties and masses and DV_CoM position in bird-line taxa.

Supplementary Data 22: Ancestral state node values for normalised dorso-ventral and cranio-caudal CoM positiions with full data set and with the pelican removed.

Supplementary Data 23: Ancestral state node values for normalised linear body segment parameters.

Supplementary Data 24: Ancestral state node values for normalised body segment masses.

Supplementary Data 25: Results of Spearman's rank correlations between normalised body segment linear properties and masses and CC_CoM position using all Ancestral State node values.

Supplementary Data 26: Results of Spearman's rank correlations between normalised body segment linear properties and masses and DV_CoM position using all Ancestral State node values.

Supplementary Data 27: Results of Spearman's rank correlations between normalised body segment linear properties and masses and CC_CoM position using Ancestral State node values 1-15.

Supplementary Data 28: Results of Spearman's rank correlations between normalised body segment linear properties and masses and DV_CoM position using Ancestral State node values between nodes 1-15.

Supplementary Data 29: Results of Spearman's rank correlations between normalised limb lengths and limb segment lengths in raw taxa values and ancestral state node values.

Supplementary Data 30: PC percentage scores using homogeneous and heterogeneous density in birds + extinct non-avian archosaur analysis, using the full data set and with the pelican removed.

Supplementary Data 31: Parameters loadings in PC analysis of linear parameters with CoM and heterogeneous density in birds + extinct non-avian archosaur analysis, using the full data set and with the pelican removed.

Supplementary Data 32: Parameters loadings in PC analysis of body segment mass with CoM and heterogeneous density in birds + extinct non-avian archosaur analysis, using the full data set and with the pelican removed.

Supplementary Data 33: Taxon scores for PCA analysis of linear parameters with CoM included and heterogeneous density in birds + extinct non-avian archosaur analysis, using the full data set and with the pelican removed.

Supplementary Data 34: Taxon scores for PCA analysis of body segment mass parameters with CoM included and heterogeneous density in birds + extinct non-avian archosaur analysis, using the full data set and with the pelican removed.

File Name: Supplementary Code 1
Description: R code, input data and phylogenetic trees for comparisons of CoM positions and body segment proportions in extant birds (results in Supplementary Data 7-17).

File Name: Supplementary Code 2
Description: R code, input data and phylogenetic tree for pANOVA comparisons of normalised body segment lengths and masses in extant birds (results in Supplementary Data 18-19).

File Name: Supplementary Code 3
Description: R code, input data and phylogenetic trees for PGLS and OLS regression of skin volume vs minimum convex hull volume in extant archosaurs and lizards (results in Supplementary Data 3-5).

File Name: Supplementary Code 4
Description: R code, input data and phylogenetic trees for ancestral state reconstructions and pPCA analyses (Supplementary Data 20-34).
