# Supplementary material for: Decoupling body shape and mass distribution in birds and their dinosaurian ancestors
Source: Nat Commun. 2023 Mar 22;14:1575. doi: 10.1038/s41467-023-37317-y (PMC10033513; doi:10.1038/s41467-023-37317-y)
Supplement: Supplementary file 4 — Supplementary Code 1-4 [file 41467_2023_37317_MOESM4_ESM.zip › SupplementaryCode_README.rtf]

README file explaining contents of SupplementaryCode.zip.Zip contains:SupplementaryCode1: R code, input data and phylogenetic trees for comparisons of CoM positions and body segment proportions in extant birds (results in Supplementary Data 7-17).SupplementaryCode2: R code, input data and phylogenetic tree for pANOVA comparisons of normalised body segment lengths and masses in extant birds (results in Supplementary Data 18-19).SupplementaryCode3: R code, input data and phylogenetic trees for PGLS and OLS regression of skin volume vs minimum convex hull volume in extant archosaurs and lizards (results in Supplementary Data 3-5).Supplementary Code4:  R code, input data and phylogenetic trees for ancestral state reconstructions and pPCA analyses (Supplementary Data 20-34)
