# Supplementary material for: Decoupling body shape and mass distribution in birds and their dinosaurian ancestors
Source: Nat Commun. 2023 Mar 22;14:1575. doi: 10.1038/s41467-023-37317-y (PMC10033513; doi:10.1038/s41467-023-37317-y)
Supplement: Supplementary file 5 — Reporting Summary [file 41467_2023_37317_MOESM5_ESM.pdf]

nature portfolio

Corresponding author(s): Karl Bates

Last updated by author(s): 09/08/2022

Reporting Summary

Nature Portfolio wishes to improve the reproducibility of the work that we publish. This form provides structure for consistency and transparency in reporting. For further information on Nature Portfolio policies, see our Editorial Policies and the Editorial Policy Checklist.

Statistics

For all statistical analyses, confirm that the following items are present in the figure legend, table legend, main text, or Methods section.

n/a	Confirmed	
☐	☒	The exact sample size (*n*) for each experimental group/condition, given as a discrete number and unit of measurement
☐	☒	A statement on whether measurements were taken from distinct samples or whether the same sample was measured repeatedly
☐	☒	The statistical test(s) used AND whether they are one- or two-sided<br>*Only common tests should be described solely by name; describe more complex techniques in the Methods section.*
☒	☐	A description of all covariates tested
☒	☐	A description of any assumptions or corrections, such as tests of normality and adjustment for multiple comparisons
☐	☒	A full description of the statistical parameters including central tendency (e.g. means) or other basic estimates (e.g. regression coefficient) AND variation (e.g. standard deviation) or associated estimates of uncertainty (e.g. confidence intervals)
☐	☒	For null hypothesis testing, the test statistic (e.g. *F*, *t*, *r*) with confidence intervals, effect sizes, degrees of freedom and *P* value noted<br>*Give P values as exact values whenever suitable.*
☒	☐	For Bayesian analysis, information on the choice of priors and Markov chain Monte Carlo settings
☒	☐	For hierarchical and complex designs, identification of the appropriate level for tests and full reporting of outcomes
☒	☐	Estimates of effect sizes (e.g. Cohen's *d*, Pearson's *r*), indicating how they were calculated

Our web collection on statistics for biologists contains articles on many of the points above.

Software and code

Policy information about availability of computer code

Data collection	As described in our methods section, CT data was segmented in Mimics (version 23) or Avizo (version 9), and the resulting 3D models were posed in Autodesk Maya (Versions 2016 and 2021).
Data analysis	All R code for our statistical analyses and associated input data have been made available with the submission. This includes R code for phylogenetic regression, anovas, ancestral state reconstruction and phylogenetic principle components analysis. These analyses were conducted using the R packages nlme v. 3.1-144, ape v. 5.3, RRPP v. 0.6.1, Paleotree v.3.3.2561, and Phytools v. 1.0-162

For manuscripts utilizing custom algorithms or software that are central to the research but not yet described in published literature, software must be made available to editors and reviewers. We strongly encourage code deposition in a community repository (e.g. GitHub). See the Nature Portfolio guidelines for submitting code & software for further information.

Data

Policy information about availability of data

All manuscripts must include a data availability statement. This statement should provide the following information, where applicable:

- Accession codes, unique identifiers, or web links for publicly available datasets
- A description of any restrictions on data availability
- For clinical datasets or third party data, please ensure that the statement adheres to our policy

3D models and numerical input data into statistical analyses and associated code are available at https://doi.org/10.17638/datacat.liverpool.ac.uk/2164. Previously

published models are available at: http://datacat.liverpool.ac.uk/310, http://dx.doi.org/10.5061/dryad.hh74n and https://osf.io/6zamj. Animal silhouettes were sourced from www.phylopic.org.

Human research participants

Policy information about studies involving human research participants and Sex and Gender in Research.

Reporting on sex and gender	N/A
Population characteristics	N/A
Recruitment	N/A
Ethics oversight	N/A

Note that full information on the approval of the study protocol must also be provided in the manuscript.

Field-specific reporting

Please select the one below that is the best fit for your research. If you are not sure, read the appropriate sections before making your selection.

☐ Life sciences ☐ Behavioural & social sciences ☒ Ecological, evolutionary & environmental sciences

For a reference copy of the document with all sections, see nature.com/documents/nr-reporting-summary-flat.pdf

Ecological, evolutionary & environmental sciences study design

All studies must disclose on these points even when the disclosure is negative.

Study description	Our analysis has two discrete components. In part 1, we use 3D models from medical image data to examine centre-of-mass (CoM) and body segment proportions in extant birds, with particular focus on the similarities and differences between birds with more hindlimb-dominated (terrestrial) locomotor habits and those with more forelimb-dominanted (volant) locomotor habits. We do this using a combination of linear models (regression models, anovas) to examine size-normalised and allometric differences. In part 2, we combine data from these birds models with data from identically constructed models of extant lizards and crocodilians to produce predictive equations that can be used to estimate body segment masses, body mass and CoM in extinct theropod dinosaurs based on 3D models of their fossil skeletons. Application of these equations to 3D models of theropods skeletons spanning basal dinosaurs to the earliest Mesozoic birds allows to reconstruct the evolution of body proportions and mass distribution along the ancestral bird-line and into modern groups, and to assess how these changes correlate with key innovations in the locomotor system (e.g. flight, crouched bipedalism).
Research sample	33 extant birds (13 'hindlimb-dominated'; 20 forelimb-dominated) spanning all major taxonomic and locomotor groupings. 17 extant lizards and crocodilians selected to provide broad phylogenetic coverage. 14 extinct taxa selected to provide phylogenetic coverage from basal dinosaurs to the earliest birds, given the availability of near-complete skeletons in the fossil record.
Sampling strategy	Our birds were selected to provide broad coverage across major orders of extant birds, and to provide sufficient numbers to investigate differences between predominantly terrestrial vs predominantly volant locomotor groups. Similarly, lizards/crocodilians were selected to provide broad coverage of major groups/body plans for inclusion in the predictive equations. 14 extinct taxa selected to provide phylogenetic coverage from basal dinosaurs to the earliest birds, given the availability of near-complete skeletons in the fossil record.
Data collection	Authors SM, KTB, JRH and ESR were primarily responsible for data collection. For extant taxa this involve primary data collection by CT animal cadavers that died of natural causes and donated by zoos and wildlife sanctuaries. These scans were supplemented with scans freely available from online data repositories and/or previous publications. 3D models of fossil taxa were derived from previous publications.
Timing and spatial scale	Not really relevant. Data was collected during the first half of SM's PhD studies and the only time dependency was the PhD itself.
Data exclusions	Not really relevant. We did model a penguin which we ultimately excluded because of its predominantly aquatic locomotion and body plan.
Reproducibility	We presented an analysis of accuracy and reproducibility of our extant medical image based models in a previous study (Macaulay et al. 2017, Journal of Anatomy) cited in our manuscript. We examine predictive accuracy and reproducibility of our predictive equations by back-applying them to our bird models, and also provide additional sensitivity tests in our convex hull restorations (see Supporting Information).
Randomization	Not relevant to the current study design.
Blinding	Not relevant to the current study design.

Did the study involve field work? ☐ Yes ☒ No

Reporting for specific materials, systems and methods

We require information from authors about some types of materials, experimental systems and methods used in many studies. Here, indicate whether each material, system or method listed is relevant to your study. If you are not sure if a list item applies to your research, read the appropriate section before selecting a response.

Materials & experimental systems

n/a	Involved in the study
☒	☐ Antibodies
☒	☐ Eukaryotic cell lines
☐	☒ Palaeontology and archaeology
☐	☒ Animals and other organisms
☒	☐ Clinical data
☒	☐ Dual use research of concern

Methods

n/a	Involved in the study
☒	☐ ChIP-seq
☒	☐ Flow cytometry
☒	☐ MRI-based neuroimaging

Palaeontology and Archaeology

Specimen provenance	All our fossil models come from previously published peer review articles, and predominantly from an article published in a Nature Group journal (Allen et al. 2013, Nature).
Specimen deposition	Our fossil analyses involve 3D models, not actual physical specimens
Dating methods	The first and last occurrence dates used in our phylogenetic statistical analyses are presented in the Supporting Information alongside the literature sources from which they were obtained.

☒ Tick this box to confirm that the raw and calibrated dates are available in the paper or in Supplementary Information.

Ethics oversight	Ethical approval was not required. Primary fossil data (the models) came from previously published papers.

Note that full information on the approval of the study protocol must also be provided in the manuscript.

Animals and other research organisms

Policy information about studies involving animals; ARRIVE guidelines recommended for reporting animal research, and Sex and Gender in Research

Laboratory animals	N/A
Wild animals	For extant taxa this involve primary data collection by CT animal cadavers that died of natural causes and donated by zoos and wildlife sanctuaries. These scans were supplemented with scans freely available from online data repositories and/or previous publications.
Reporting on sex	We have no information on the sex of the animals used.
Field-collected samples	N/A
Ethics oversight	No ethical approval was required as animals were either donated by zoos/wildlife sanctuaries after death by natural causes, scanned for us by other collaborators or came from previously published studies.

Note that full information on the approval of the study protocol must also be provided in the manuscript.
